# Comparative genomics of 10 new *Caenorhabditis* species

**DOI:** 10.1002/evl3.110

**Published:** 2019-04-02

**Authors:** Lewis Stevens, Marie‐Anne Félix, Toni Beltran, Christian Braendle, Carlos Caurcel, Sarah Fausett, David Fitch, Lise Frézal, Charlie Gosse, Taniya Kaur, Karin Kiontke, Matthew D. Newton, Luke M. Noble, Aurélien Richaud, Matthew V. Rockman, Walter Sudhaus, Mark Blaxter

**Affiliations:** ^1^ Institute of Evolutionary Biology, Ashworth Laboratories, School of Biological Sciences University of Edinburgh Edinburgh EH9 3JT United Kingdom; ^2^ Institut de Biologie de l'Ecole Normale Supérieure, Centre National de la Recherche Scientifique, Institut National de la Santé et de la Recherche Médicale, École Normale Supérieure Paris Sciences et Lettres 75005 Paris France; ^3^ MRC London Institute of Medical Sciences London W12 0NN United Kingdom; ^4^ Université Côte d'Azur, Centre National de la Recherche Scientifique, Inserm Institute of Biology Valrose 06108 Nice France; ^5^ Department of Biology New York University New York New York 10003; ^6^ Center for Genomics and Systems Biology, Department of Biology New York University New York New York 10003; ^7^ Molecular Virology, Department of Medicine Imperial College London Du Cane Road London W12 0NN United Kingdom; ^8^ Institut für Biologie/Zoologie Freie Universität Berlin Berlin D‐14195 Germany

**Keywords:** *C. elegans*, genomics, phylogenomics, morphology, species description

## Abstract

The nematode *Caenorhabditis elegans* has been central to the understanding of metazoan biology. However, *C. elegans* is but one species among millions and the significance of this important model organism will only be fully revealed if it is placed in a rich evolutionary context. Global sampling efforts have led to the discovery of over 50 putative species from the genus *Caenorhabditis*, many of which await formal species description. Here, we present species descriptions for 10 new *Caenorhabditis* species. We also present draft genome sequences for nine of these new species, along with a transcriptome assembly for one. We exploit these whole‐genome data to reconstruct the *Caenorhabditis* phylogeny and use this phylogenetic tree to dissect the evolution of morphology in the genus. We reveal extensive variation in genome size and investigate the molecular processes that underlie this variation. We show unexpected complexity in the evolutionary history of key developmental pathway genes. These new species and the associated genomic resources will be essential in our attempts to understand the evolutionary origins of the *C. elegans* model.

**Impact summary**

*Caenorhabditis elegans* is a tiny, free‐living nematode, or roundworm, which is been used extensively in biological research. Recent years have seen a focused effort to discover new species that are closely related to *C. elegans* in the hope that they will help us understand how this important model organism evolved. We present formal species descriptions and names for 10 new species of *Caenorhabditis* isolated from across the world. We sequenced the genomes and transcriptomes of each new species and use the data to reconstruct the evolutionary history of these and other *Caenorhabditis* species. We demonstrate the utility of these new species and their associated data by analyzing the evolution of morphology, the evolution of important developmental genes, and the evolution of genome size. These new resources will be essential to our attempts to understand the evolutionary origins of this important nematode.

*Caenorhabditis elegans* has become one of the preeminent model organisms in modern biology, but only recently have we started to understand its natural ecology and evolutionary history (Félix & Braendle 2010). Studying *C. elegans* within the context of its wild ecology and alongside its closest relatives will provide an important evolutionary context for the particular systems analyzed within the laboratory. A large collection of wild‐caught strains of *C. elegans* is now available for comparative exploration of natural variation (Cook et al. 2016, 2017), and ecological interactions are being investigated through co‐analysis of nematodes and microbial associates from natural systems (Schulenburg & Félix 2017). In parallel, the last decade has seen a focused search for new species in the genus *Caenorhabditis*.

The discovery of new *Caenorhabditis* species was, for many years, hindered by a poor understanding of the natural ecology of these nematodes (Félix & Braendle 2010). Surveys of natural populations of *C. elegans* and *Caenorhabditis briggsae* revealed that, rather than being “soil nematodes,” *Caenorhabditis* species thrive in microbe‐rich environments, such as rotting fruits, flowers, and stems (Kiontke *et al*. 2011; Félix & Duveau 2012; Félix et al. 2014; Ferrari et al. 2017). This new understanding, combined with extensive worldwide sampling efforts, has led to the discovery of more than 50 species (Kiontke et al. 2011; Félix et al. 2014; Ferrari et al. 2017; MAF, LF, MVR, CB, unpublished; John Wang, Michael Ailion, Erik Andersen, Asher Cutter, pers. comm.) of *Caenorhabditis*, many of which await formal species description.

Although morphology remains fundamental to species diagnosis, closely related *Caenorhabditis* species are often morphologically very similar (Sudhaus & Kiontke 2007) and many morphological characters are homoplasious within the genus (Kiontke et al. 2011). This has motivated the use of mating tests for species definition and comparisons of molecular sequences such as ribosomal DNA (rDNA) or internal transcribed spacer (ITS) sequences, in addition to morphology, for species diagnosis (Kiontke et al. 2011; Félix et al. 2014). These molecular sequences have also enabled a reconstruction of the *Caenorhabditis* phylogeny (Cho et al. 2004; Kiontke et al. 2004, 2011). Recently, whole‐genome data have been exploited for phylogenomic analysis (Slos et al. 2017).

Genome sequences of species closely related to *C. elegans* have furthered our understanding of *C. elegans* biology and revealed insights into the evolutionary forces that have shaped its genome. The publication of the *C. briggsae* genome in 2003 enabled the first comparative genomics studies of *Caenorhabditis*, which revealed an unusually high rate of intrachromosomal rearrangement but comparatively rare interchromosomal rearrangement (Stein et al. 2003). Additional genome sequences have been published from species across the genus (Mortazavi et al. 2010; Fierst et al. 2015; Slos et al. 2017; Kanzaki et al. 2018; Ren et al. 2018; Yin et al. 2018). Comparisons between genomes of hermaphroditic species such as *C. elegans* and their outcrossing relatives have exposed the genomic consequences of a switch in reproductive mode from gonochorism to autogamous hermaphroditism, including changes in overall genome structure, gene structure, and protein‐coding gene content (Thomas et al. 2012; Fierst et al. 2015; Kanzaki et al. 2018; Yin et al. 2018).

Here, we use mating tests, accompanied by molecular and morphological analyses, to characterize and describe 10 new *Caenorhabditis* species isolated from across the world. We present draft genome sequences for nine of the 10 new species, and a transcriptome assembly for one, and use the data to reconstruct the *Caenorhabditis* phylogeny. By studying morphology in the context of this phylogenetic tree, we find further examples of homoplasious morphological characters in the genus. We use the genome sequences to study variation in genome size and the evolution of genes involved in a key developmental pathway. These new species and their draft genome sequences will become an important resource for the growing number of evolutionary biologists who use *Caenorhabditis* in their research.

## Results

### TEN NEW SPECIES DECLARATIONS

By sampling a variety of substrates in diverse geographic locations, we found 10 new species of *Caenorhabditis*, most of them from rotting fruit. While initial selection of isolates for further analysis was based on morphological or molecular assignment to *Caenorhabditis*, as argued and implemented in Félix et al. (2014), the justification for raising species is based on a biological species concept that can be easily implemented for these culturable nematodes. Thus, for each new isolate, we attempted crosses with previously described species in culture that have the most similar ribosomal RNA cistron internal transcribed spacer 2 (rDNA ITS2) sequence (Table 1 or Table S1). The results of the crosses are shown in Table S2. For two putative species, *Caenorhabditis parvicauda* sp. n. and *Caenorhabditis uteleia* sp. n., that also had striking morphological novelty (see below), no alignment of the rDNA ITS2 region could be obtained with default parameters in NCBI nucleotide BLAST (word size 28, gap penalty 1,2, gap costs 0,2.5). We considered these highly divergent from any known species and did not perform mating tests. Based on the phylogenetic relationships detailed below, the 10 species all belong to the clade of species whose most basally branching member is *Caenorhabditis monodelphis* (Slos et al. 2017) and are therefore in the genus *Caenorhabditis* (Osche 1952; Dougherty 1953).

**Table 1:** *Caenorhabditis* species names, reference isolates and sequenced strains

*Caenorhabditis* species name	Temp. species number	Abbreviation	Reference isolate	Sequenced strain (inbreeding rounds)	Genome accession number	rDNA accession number(s)
*Caenorhabditis parvicauda* sp. n.	*C*. sp. 21	*Cpv*	NIC134	NIC534 (25x)	PRJEB12595	MH800325
*Caenorhabditis zanzibari* sp. n.	*C*. sp. 26	*Cza*	JU2161	JU2190 (26x)	PRJEB12596	MH809973, MH809941, MH809969
*Caenorhabditis panamensis* sp. n.	*C*. sp. 28	*Cpa*	QG702	QG2080 (25x)	PRJEB28259	MH809974, MH809942, MH809970
*Caenorhabditis becei* sp. n.	*C*. sp. 29	*Cbe*	QG704	QG2083 (25x)	PRJEB28243	MH809975, MH809943, MH809971
*Caenorhabditis uteleia* sp. n.	*C*. sp. 31	*Cut*	JU2469	JU2585 (25x)	PRJEB12600	MH800326
*Caenorhabditis sulstoni* sp. n.	*C*. sp. 32	*Csu*	SB454	JU2788 (25x)	PRJEB12601	MH800333
*Caenorhabditis quiockensis* sp. n.	*C*. sp. 38	*Cqu*	JU2745	JU2809 (25x)	PRJEB11354	MH800334
*Caenorhabditis waitukubuli* sp. n.	*C*. sp. 39	*Cwt*	NIC564	NIC564 (isofemale)	PRJEB12602	MH800335
*Caenorhabditis tribulationis* sp. n.	*C*. sp. 40	*Ctb*	JU2774	JU2818 (25x)	PRJEB12608	MH809976, MH809944, MH809972,
*Caenorhabditis vivipara* sp. n.	*C*. sp. 43	*Cvv*	NIC1070	NIC1070	PRJEB12605	MH800336

Specific name referents were derived as follows: 21: Reduced tail (in the male). 26: First isolated in Zanzibar. 28: First isolated in Panama. 29: First isolated on Barro Colorado Island, Panama. 31: U‐shape male tail. 32: In honor of John Sulston. 38: First isolated on Quiock river trail, Guadeloupe. 39: First isolated on the island of Dominique, in the local native American language. 40: First isolated in the region of Cape Tribulation, Australia. 43: Viviparous.

The formal descriptions of each species can be found in Document S1. We describe the following new species (see Table 1 for the correspondence with the informal numbering system used to refer to the species in previous publications):

- *Caenorhabditis becei* sp. n.
- *Caenorhabditis panamensis* sp. n.
- *Caenorhabditis parvicauda* sp. n.
- *Caenorhabditis quiockensis* sp. n.
- *Caenorhabditis sulstoni* sp. n.
- *Caenorhabditis tribulationis* sp. n.
- *Caenorhabditis uteleia* sp. n.
- *Caenorhabditis vivipara* sp. n.
- *Caenorhabditis waitukubuli* sp. n.
- *Caenorhabditis zanzibari* sp. n.

### GENOME SEQUENCES OF NINE NEW *CAENORHABDITIS* SPECIES

We sequenced the genomes of all newly described species to high coverage (100–350×) using short‐read Illumina technology. After identifying and removing reads from nontarget organisms, we generated draft assemblies for each species, employing heterozygosity‐aware assembly approaches where necessary. Draft assemblies were scaffolded using assembled transcripts or long‐insert (or “mate‐pair”) data, where available. The sequence data generated for *Caenorhabditis vivipara* were not of sufficient quality to generate a reference assembly and are not discussed further. All assemblies have been submitted to DDBJ/ENA/GenBank (Table 1).

Assembly span indicated substantial variation in genome size among species (Table 2). At 65.1 Mb, the genome of *Caenorhabditis sulstoni* is the smallest *Caenorhabditis* genome published thus far, and nearly 35 Mbp smaller than the *C. elegans* genome. For all species other than *C. waitukubuli* (see below), assembly spans were consistent with semi‐independent estimates based on kmer spectra analysis (Fig. S1). The contiguity of the resulting assemblies was highly variable. The assemblies of *Caenorhabditis becei* and *C. panamensis*, which were scaffolded with long‐insert data, are the most contiguous, with N50 lengths of 487 and 768 kbp, respectively. The assembly of *Caenorhabditis waitukubuli* is the least contiguous, with an N50 length of 15.1 kbp. Kmer spectrum analysis (Fig. S2) indicated extensive heterozygosity present in the genome of this strain, which has not been fully collapsed during assembly. The proportion of undetermined bases (i.e., gaps denoted as Ns) was low in all cases. Despite considerable differences in assembly contiguity, Benchmarking Universal Single‐Copy Orthologs (BUSCO) and Core Eukaryotic Genes Mapping Approach (CEGMA) indicated that all assemblies were of high gene‐level completeness.

**Table 2:** Genome assembly and annotation metrics for nine species of *Caenorhabditis*

Species	Species number	Sequenced strain	Assembly span (Mbp)	Scaffold count (n)	N50 (kbp)	Ns%	BUSCO genome complete/fragmented %	Protein‐coding gene count (*n*)	BUSCO proteome complete/fragmented %
*C. parvicauda*	21	NIC534	93.7	5,719	44.4	1.11	89.5/5	16,412	89.9/6.4
*C. zanzibari*	26	JU2190	101.1	3,128	91.3	0.22	98.2/1.2	22,198	98.6/1
*C. panamensis*	28	QG2080	79.0	986	487.2†	1.16	97.3/1.8	17,134*	91.9/3.6
*C. becei*	29	QG2083	87.9	1,567	767.5†	1.74	97.5/1.4	18,669*	91.9/3.9
*C. uteleia*	31	JU2585	104.0	3,222	177.0	0.99	96/3.1	27,614	96.1/3.1
*C. sulstoni*	32	JU2788	65.1	2,044	136.7	0.59	97.9/1	18,192	95.3/3.3
*C. quiockensis*	38	JU2809	100.4	4,890	139.4	0.09	96.1/3.2	22,278	95.7/3.4
*C. waitukubuli*	39	NIC564	91.4	21,203	15.1	1.27	92.5/4.8	30,089	92.7/5.5
*C. tribulationis*	40	JU2818	101.2	3,276	224.5	0.15	97.7/1.1	24,787	97.9/1.4
*C. elegans*	‐	N2	100.2	7	17,493.8	0.00	98.4/1.0‡	20,094	99.7/0.3‡

^*^RNA‐seq data were not generated and therefore not used to guide gene prediction.

^†^long‐insert (or “mate‐pair”) data were used during assembly. BUSCO version 3.0.2 was used with the ‘Nematoda_odb9’ dataset for both genome and proteome completeness assessment. Scaffolds shorter than 500 bp were discarded prior to annotation. Wormbase version WS264 of the *C. elegans* genome was used (Lee *et al*. 2018).

^‡^BUSCO does not find core genes in the complete *C. elegans* N2 genome. More detailed metrics are presented in Table S3.

RNA‐seq data were used to guide gene prediction for all species except *C. becei* and *C. panamensis*. The number of protein‐coding genes predicted in each assembly varied considerably. The genome of *C. parvicauda* has the fewest predicted genes, at 16,412. This is likely an underestimate of the true gene number, as ∼5% of BUSCO genes were absent from the assembly. *C. waitukubuli* has 30,089 predicted protein‐coding genes. Overall 19.8% of BUSCO genes found in this assembly were present in multiple copies, suggesting that this high gene number is an artifact arising from regions of uncollapsed heterozygosity present in the assembly. The lack of RNA‐seq data for *C. becei* and *C. panamensis* resulted in less complete gene sets, with a larger percentage of BUSCO genes missing from the gene sets (4.5% and 4.2%, respectively) than from the draft assemblies (0.9% and 1.1%, respectively).

### PHYLOGENETIC RELATIONSHIPS WITHIN THE GENUS *CAENORHABDITIS*

Previous analyses of *Caenorhabditis* phylogeny have used morphology or small numbers of loci and have defined subgeneric groups of taxa (Kiontke et al. 2011): the *Elegans* supergroup, which contains the *Japonica* and *Elegans* groups, and the *Drosophilae* supergroup, which contains the *Drosophilae* and *Angaria* groups. We exploited our new and existing genomic data to re‐examine the phylogenetic structure of *Caenorhabditis*. We performed orthology clustering of 781,865 protein sequences predicted from the genomes and transcriptomes of all 10 newly described species, 22 other *Caenorhabditis* species (*C. elegans* Sequencing Consortium 1998; Stein et al. 2003; Mortazavi et al. 2010; Kanzaki et al. 2018; Yin et al. 2018), and from the outgroup taxon *Diploscapter coronatus* (Hiraki et al. 2017). We identified 1988 single‐copy orthologues, each of which was present in at least 27 of the 33 taxa, and aligned their amino‐acid sequences. We performed maximum likelihood (ML) and Bayesian inference (BI) analyses on a concatenated alignment of these loci. We also employed a supertree approach by estimating gene trees for all single‐copy loci using ML analysis and providing the resulting topologies to ASTRAL‐III (Mirarab & Warnow 2015) to estimate the species tree.

**Figure 1:**
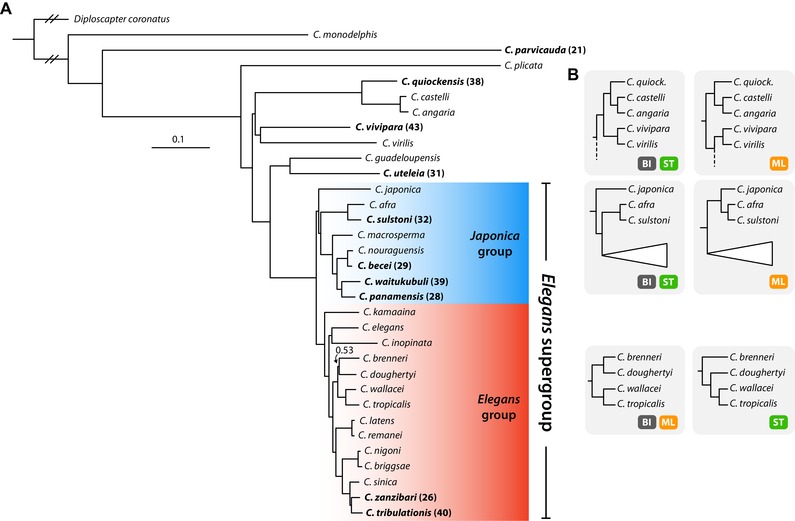
Phylogenetic relationships of 32 *Caenorhabditis* species and *D. coronatus*. **(A)** Phylogeny inferred using Bayesian inference with the CAT‐GTR+Γ substitution model. Species described here are highlighted in bold, with previous species numbers in parentheses. Bayesian posterior probabilities are 1.0 unless noted as branch annotations. Scale is in substitutions per site. **(B)** Alternative hypotheses and support from each analysis approach. ML, Maximum likelihood inference using GTR+Γ substitution model; BI, Bayesian inference using the CAT‐GTR+Γ substitution model; ST, Supertree approach, using gene trees as input (substitution model selected automatically for each alignment).

The three analyses yielded highly congruent, well‐supported topologies that displayed very few inconsistencies, discussed below (Fig. 1; Fig. S3–5). The majority of relationships, including the monophyly of both the *Elegans* and *Japonica* groups, were recovered with maximal support (bootstrap support of 100 and Bayesian posterior probability values of 1) regardless of method. All approaches recovered a clade of *C. guadeloupensis* + *C. uteleia* as sister to the *Elegans* supergroup. We found *C. parvicauda* to be the second‐most basally arising species in the genus. We recovered *C. sulstoni, C. becei, C. waitukubuli*, and *C. panamensis* as members of the *Japonica* group. The sister taxa *C. zanzibari* and *C. tribulationis* were placed as members of the *Elegans* group, being most closely related to *C. sinica*. We recovered *C. quiockensis* as sister to *C. angaria* + *C. castelli*.

Three relationships, which tended to have lower‐than‐maximal support, were inconsistent across methods (Fig. 1B). Both the supertree approach and BI recovered the *C. vivipara* + *C. virilis* group as sister to the clade containing *C. quiockensis, C. castelli*, and *C. angaria*, while ML analysis recovered this group as sister to the group consisting of the sister taxa *C. guadeloupensi*s + *C. uteleia* and the *Elegans* supergroup. Second, the placement of *C. japonica* as sister to other members of the *Japonica* group was recovered by both the supertree approach and by BI, whereas ML analysis recovered *C. japonica* as sister to *C. afra* + *C. sulstoni*. Finally, BI and ML analysis recovered *C. brenneri* and *C. doughertyi* as sister taxa, whereas the supertree approach recovered *C. brenneri* as sister to the clade containing *C. doughertyi*, *C. wallacei*, and *C. tropicalis*.

### MORPHOLOGICAL NOVELTY IN *CAENORHABDITIS PARVICAUDA* SP. N. AND *CAENORHABDITIS UTELEIA* SP. N

**Figure 2:**
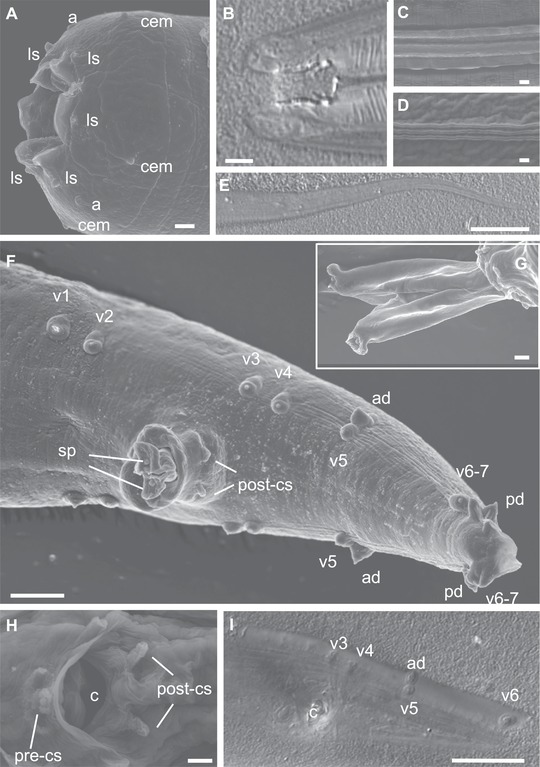
Morphology of *Caenorhabditis parvicauda* sp. n. by scanning electron microscopy (SEM) and Nomarski optics (DIC). **(A and B)** Mouth of an adult male (A, SEM; B, DIC). **(C)** Cuticular lateral ridges of a dauer juvenile (SEM). **(D)** Cuticular lateral ridges of an adult male (SEM). **(E)** Female tail (DIC). **(F)** Male tail, ventro‐lateral view (SEM). **(G)** Genital opening with extruded spicules. **(H)** Male genital opening. **(I)** Male tail in ventral view (DIC). Anterior is to the left in (A, B, E–I). The animals are from strain JU2070. a, amphid; ad, anterior dorsal papilla; c, cloaca; cem, male cephalic sensillum (absent in females); ls, labial sensillum; pd, posterior dorsal papilla; pre/post‐cs, pre/post cloacal sensillum; v1, etc.: ventral papilla 1, etc.; sp, spicule. Scale bars: 1 μm, except in (E, F, and I): 5 μm. See also Figure S6.

**Figure 3:**
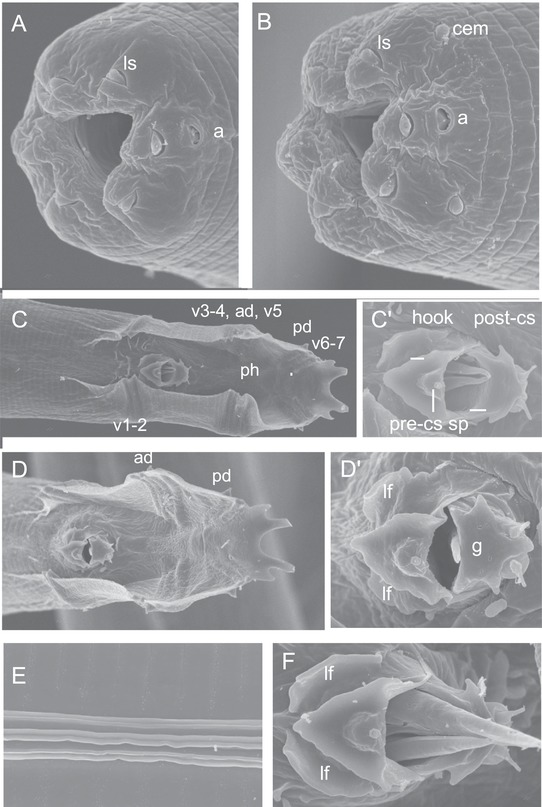
Scanning electron microscopy of *Caenorhabditis uteleia* sp. n. **(A)** Mouth of an adult female. **(B)** Mouth of an adult male. **(C and D)** Male tail in ventral view. **(C’ and D’)** Higher magnification of the corresponding male genital openings. **(E)** Cuticular lateral ridges, adult male. **(F)** Male genital opening. The animals are from strain JU2469. Anterior is to the left. a, amphid; cem, male cephalic sensillum (absent in females); lf, lateral fold on either side of the hook; ls, labial sensillum; r1, etc., ray 1, etc.; ph, phasmid; sp, spicule; pre/post‐cs, pre/post cloacal sensillum; g, posterior end of the gubernaculum. Bars: 1 μm, except in (C and D): 5 μm.

**Figure 4:**
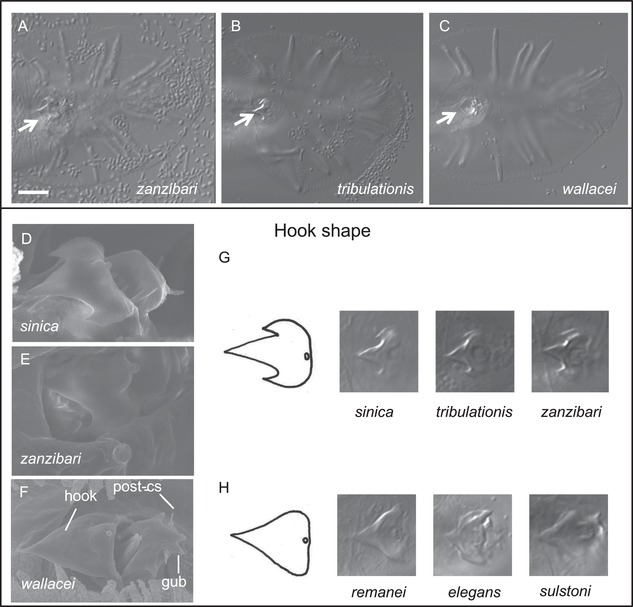
Male hook shape in *Elegans* group species. **(A–C)** Ventral views of the male tail of *Caenorhabditis zanzibari* strain JU2161 (A), *C. tribulationis* strain JU2774 (B), and *C. wallacei* JU1873 (C), in Nomarski optics. The arrow points to the precloacal hook. Bar: 10 μm. **(D–F)** Scanning electron micrographs of the hooks and post‐cloacal sensilla (post‐cs) in *C. sinica* JU727 (D), *C. zanzibari* JU2161 (E), and *C. wallacei* JU1873 (F). “gub”: forked posterior end of gubernaculum. **G,H**: Drawings of hook shape. (G) Refers to the trilobed shape in *C. sinica*, *C. tribulationis*, and *C. zanzibari* sp. n, while (H) refers to the simpler shape in most other *Elegans* supergroup species such as *C. wallacei*, *C. elegans*, *C. remanei*, and *C. sulstoni*.

Morphological features of *C. parvicauda* are displayed in Fig. 2. Most striking is the absence of a fan in the tail of *C. parvicauda* adult males (Fig. 2F, I, Fig. S6; compare with Figs. 3C,D and 4A) and a left–right asymmetry in the locations of caudal papillae. The caudal papillae include two pairs anterior to the cloaca (v1, v2) followed by groups of two (v3, v4), two (v5 and ad), and three pairs (v6, v7, and pd [posterior dorsal]) as seen in Fig. 2F, I and Fig. S6. Papillae ad (anterior dorsal) and v5 are both open to the outside. The positions of the dorsal papillae ad (anterior dorsal) and pd generally differ between the right and left sides at two levels. First, whereas papilla v5 has a similar anterior–posterior position on the right and left sides, papilla ad is generally anterior to v5 on the right side and at the same position or posterior to v5 on the left side (Fig. 2F, I and Fig. S6). Second, while papilla pd has a similar anterior–posterior position on the right and left sides, ventral papillae v6 and v7 are generally located posterior to it on the right side and anterior on the left side (Fig. 2I and Fig. S6). The spicules are thick, with a complex tip (Fig. 2G). Simple pre‐ and post‐cloacal sensilla can be seen (Fig. 2H). The males mate in a spiral position. This left–right asymmetry is highly unusual. The outer side of the mouth of *C. parvicauda* is endowed with the usual set of sensory organs disposed in a concentric manner, namely six labial sensillae, two amphids, and four male‐specific cephalic sensillae (Fig. 2A). The buccal cavity is short compared to most other *Caenorhabditis* (Sudhaus & Kiontke 1996), with two teeth at the base. The pharyngeal sleeve extends anteriorly to half of the buccal cavity (Fig. 2B). Three cuticular ridges can be seen in the lateral field of adults of both sexes (Fig. 2C, D). The adult female tail end is long and thin (Fig. 2E).

The morphology of *C. uteleia* is remarkable for the contour of its male tail fan (Fig. 3). The posterior margin shows a distinctive complex shape with one large central valley and two smaller ones on the left and right sides (Fig. 3C, D). The fan is well developed, opened anteriorly, and shows a smooth, unserrated margin. One group of two pairs of rays is found anterior to the cloaca, followed by two groups of four and three pairs. The dorsal rays are in antero‐posterior positions 5 and 7. The pre‐cloacal sensillum is on a hook, itself in between two characteristic lateral folds (Fig. 3C’, D’, F show the hook, the gubernaculum ventral end with two lateral ears, and the pair of post‐cloacal sensilla). The spicule tip is pointed (Fig. 3C’, F).

### MORPHOLOGICAL CHARACTER EVOLUTION

What are the implications of the new species and the new phylogenetic tree for character evolution in the genus? Character evolution in *Caenorhabditis* was first studied in Sudhaus & Kiontke (1996) based on a morphology‐based phylogenetic tree. Kiontke et al. (2011) and Félix et al. (2014) mapped morphology onto a previous six‐gene phylogenetic tree and recently, Slos et al. (2017) discussed the stem species pattern using a morphological comparison with the sister group Protoscapter.

**Figure 5:**
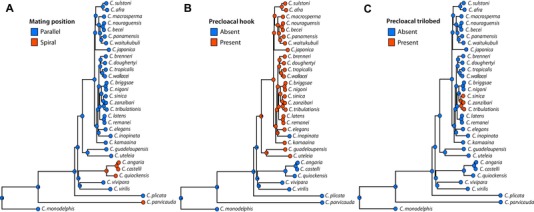
Ancestral state reconstruction of precloacal hook morphology and mating position. **(A)** Mating position. **(B)** Precloacal lip in the shape of a hook (with a pointed ventral tip). (**C)** Precloacal lip in the shape of a trilobed hook. Ancestral state reconstruction was performed by generating 1000 stochastic character maps of each morphological character on the phylogenetic tree in Figure 1A using the equal rates model of evolution. Pie charts on internal nodes represent posterior probabilities of ancestral states.

Starting from the base of the genus, defined by the placement of the sister taxon Protoscapter (Slos et al. 2017), containing *Diploscapter*, we now have as successive branches *C. monodelphis* (likely to be associated with the presently unavailable *Caenorhabditis sonorae*; Slos et al. 2017), then *C. parvicauda* and then *C. plicata*. Species in these three branches share characters that are ancestral in the genus (plesiomorphous), such as the absence of a hook. All four species differ greatly in morphology and show private characters and combinations thereof. *C. parvicauda* is unusual for *Caenorhabditis* because the male tail does not include an extended fan and papilla v5 does not appear broader than the others. However, in contrast to *C. monodelphis*, *C. parvicauda* displays some characters that are shared with other *Caenorhabditis* species, such as three lateral cuticular ridges. *C. parvicauda* also shares some characters with some other species that are likely homoplastic. For example, *C. parvicauda* mate in a spiral fashion on an agar plate, as do species of the *Angaria* group (Fig. 5A).

*C. quiockensis* is typical for the *Angaria* group (*C. angaria*, *C. castelli*; Kiontke et al. 2011; Félix et al. 2014) in displaying spiral mating and having a distinctive male tail morphology (Fig. S7A–C). The fan has an oval shape in ventral view and is open anteriorly. Rays 4 (= ad) and 7 (= pd) open dorsally to the velum.

*C. vivipara* is distinct in its viviparity. In laboratory conditions, the females do not lay embryonated eggs (however, we cannot rule out that they lay embryos in some environment). Instead, L1 larvae exit their mother's body through the vulva, as, for example, in the genus *Panagrellus* in family Panagrolaimidae (Andrássy 1983; Félix et al. 2018). In the closely related species *C. virilis*, females lay late‐stage embryonated eggs compared to most *Caenorhabditis* species (which accumulate few embryos in their uteri) but are not viviparous in standard laboratory conditions. Like *C. virilis*, however, *C. vivipara* has a wide and heart‐shaped male fan, similar to the male fans of species in the *Elegans* supergroup yet with no terminal notch (compare Figs. S7E and S8).

*C. uteleia* has a previously unknown combination of characters that do not all match its most closely related known species *C. guadeloupensis* (Kiontke et al. 2011; Félix et al. 2014). For example, the male fan of *C. uteleia* is open and unserrated on the anterior side as observed in many species in the *Drosophilae* supergroup and in basally branching species. Conversely, the ad ray is in the fifth position in *Elegans* supergroup species and basally branching species such as *C. monodelphis* (Slos et al. 2017) but not in *C. guadeloupensis*. The latter, such as *C. plicata*, *C. drosophilae*, the *Angaria* group, *C. virilis*, and *C. vivipara*, shows a dorsal ray in anteroposterior position 4 (Kiontke et al. 2011; Félix et al. 2014; present data). Thus, these characters remain homoplastic in the new phylogenetic tree (Fig. S9) and will most likely remain so. Finally, the new clade that includes *C. guadeloupensis*, *C. uteleia*, and the *Japonica* + *Elegans* groups is distinguished by the presence of a hook bearing the pre‐cloacal sensillum on the anterior margin of the cloaca (Fig. 5B). This character also appears in *C. portoensis* but not in *C. virilis* (Kiontke et al. 2011; Félix et al. 2014) or *C. vivipara* (Fig. S7F, G), and thus it will be important to affirm the position of *C. portoensis* (currently resolved in a clade including *C. virilis;* Kiontke et al. 2011; Félix et al. 2014) to define how many times this character has evolved.

In contrast, the clade including the *Elegans* and *Japonica* groups displays little variation except for the tropical fig species *C. inopinata* (Kanzaki et al. 2018). One character variation that is unique to the *Elegans* group is the independent evolution of hermaphroditism in three species (Kiontke et al. 2011; Félix et al. 2014). The present work does not add new hermaphroditic species, as all 10 new species reproduce through females and males. However, using the large set of species studied here including six new species in the *Elegans* and *Japonica* groups, we distinguished characters in the male tail that vary in either the *Elegans* or *Japonica* group. Interestingly, none of these characters correlate with reproductive mode. Within the *Elegans* group, the small clade containing *C. sinica*, *C. zanzibari*, and *C. tribulationis* displays a characteristic shape of the male hook with three marked lobes (Fig. 4). The hook of other species of the *Elegans* supergroup such as *C. elegans* displays a single lobe. The complex shape of the hook as first described for *C. sinica* as a specific character (Huang et al. 2014) now constitutes a clear apomorphy (derived character), with no pattern of convergent evolution (Fig. 5C).

In the *Japonica* group, ray v4 is shorter in the clade including *C. becei*, *C. waitukubuli*, *C. panamensis*, and *C. nouraguensis*, compared to *C. sulstoni*, *C. afra*, *C. elegans*, and many other species in which ray v4 displays a similar length to the ad (anterior dorsal) ray (see also drawings in Kiontke et al. (2011). Ray v4 is also short in *C. japonica* (Kiontke et al. 2002), but longer in *C. macrosperma* JU1857, which implies events of homoplasy in any of the configurations of the phylogenetic tree for this latter species (Fig. 1).

Kiontke et al. (2011) and Félix et al. (2014) noted that the ventral tip of the spicules was broad and complex outside the *Elegans* supergroup as well as in *C. japonica* and *C. afra*. The ventral tip of the spicules is wide in *C. afra* as well as its sister species *C. sulstoni* (Fig. S10). It also seems broader and bent at an angle in *C. becei*, more so than in *C. panamensis* or *C. waitukubuli* (Fig. S10). This character thus varies in the *Japonica* group, while the tip remains pointed in the *Elegans* group, and given the present phylogeny, some homoplasy must also be present in this character.

### GENOME SIZE VARIATION IN *CAENORHABDITIS*

The molecular mechanisms and evolutionary forces that underlie interspecific variation in genome size remain poorly understood. Hermaphroditism has evolved three times independently in *Caenorhabditis* (Kiontke et al. 2011), and this switch in reproductive mode is hypothesized to impact genome size and content (Lynch & Conery 2003). Previous studies have revealed that genomes and transcriptomes of hermaphroditic species are smaller than those of their outcrossing relatives (Thomas et al. 2012; Fierst et al. 2015). A comparison of the genomes of the closely related sister taxa *C. nigoni* and *C. briggsae* revealed that the genome of *C. briggsae* has undergone extensive contraction after the evolution of hermaphroditism, which has largely been driven by loss of genes with male‐biased expression (Yin et al. 2018).

**Figure 6:**
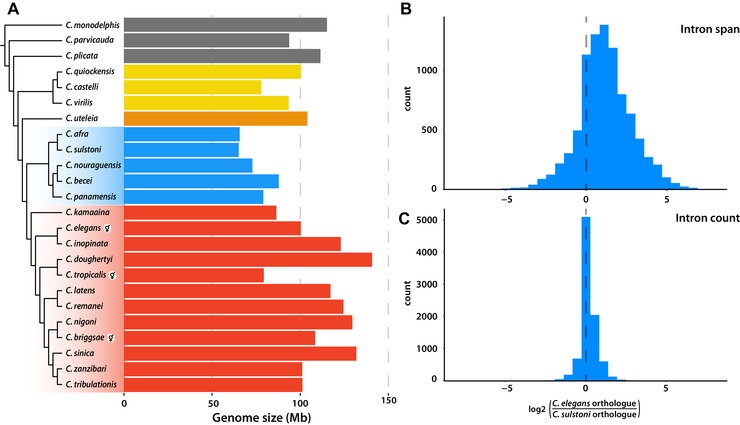
Genome size and gene structure variation in *Caenorhabditis*. **(A)** Genome size variation in the context of the *Caenorhabditis* phylogeny. Hermaphroditic species are highlighted. Phylogenetic tree is based on Figure 1A, with major clades highlighted. **(B)** Histogram of the log_2_‐transformed ratio of intron span in 8954 genes in *C. elegans* compared to their orthologues in *C. sulstoni*. **(C)** Histogram of the log_2_‐transformed ratio of intron count in 8954 genes in *C. elegans* compared to their orthologues in *C. sulstoni*.

To study genome size variation in the genus, we first identified genome assemblies that had an excess of single‐copy loci present in two or more copies. The presence of such duplicates suggests that an assembly includes significant uncollapsed haploid allelic segments, and thus represents an inflated genome size estimate. We thus excluded five genomes from subsequent analyses. By comparing genome size of the remaining species within the context of the *Caenorhabditis* phylogeny, we explored the extensive variation in genome size, ranging from 65 Mb (the genomes of the sister taxa *C. afra* and *C. sulstoni*) to 140 Mb (the genome of *C. doughertyi*; Fig. 6A). The genomes of all three hermaphroditic species (*C. briggsae, C. elegans*, and *C. tropicalis*) are smaller than their most closely related outcrossing relatives (*C. nigoni*, *C. inopinata*, and *C. doughertyi*, respectively). We note that the genome of *C. wallacei*, the closest known relative of *C. tropicalis*, is not yet publicly available and was not included in this analysis.

To investigate the unusually small genome of *C. sulstoni*, we compared the content of its genome with that of *C. elegans* (Table S5). Relative to *C. elegans*, *C. sulstoni* has 2170 fewer genes, 1694 (78%) of which are due to a reduction in gene family size. By comparing gene structure in 8,954 single‐copy orthologues between *C. sulstoni* and *C. elegans*, we found that genes in *C. sulstoni* are on average shorter, largely because introns have undergone contraction (Fig. 6B). In contrast, intron–exon structure appears to be largely conserved, with limited evidence of decreased intron count in *C. sulstoni* (Fig. 6C)*. C. sulstoni* also had a reduction in mean intergenic distance (578 bp vs. 1047 bp in *C. elegans*) and in estimated repeat content (12% vs. 18% in *C. elegans*).

To explain genome size variation across the genus while taking species’ relationships into account, we used phylogenetic generalized least squares (PGLS) analysis of genome size against protein‐coding gene number, intron count, intron size, intergenic distance, and repeat content (Fig. S11; Table S6). Only protein‐coding gene number and proportion of repetitive DNA were significantly positively correlated with genome size (Figs. S11A and S11B).

### THE EVOLUTION OF NOTCH/LIN‐12 SIGNALLING PROTEINS IN *CAENORHABDITIS*

The new genome data and the resolved phylogeny permit detailed examination of the origins and diversification of genes and gene families. Developmental genetics in *C. elegans* has identified many genetic systems critical across Metazoa, but has also identified idiosyncrasies that are not shared. As an example of the power of these new data, we examine Notch signaling.

Notch signaling is a highly conserved intercellular signaling pathway involved in an array of cell fate decisions during animal development (Artavanis‐Tsakonas et al. 1999). Basic components of this pathway were characterized in *C. elegans* at the same time as in *Drosophila melanogaster* (Greenwald 1985, 2005). Central to this pathway are the Notch receptors (Greenwald 1985; Yochem et al. 1988; Yochem & Greenwald 1989). These transmembrane proteins bind extracellular ligands of the Delta/Serrate/LAG‐2 (DSL) family. Ligand binding results in cleavage and nuclear translocation of an intracellular domain that associates with transcription factors to influence the expression of genes involved in cell‐fate decisions (Greenwald 2005). *C. elegans* possesses two Notch‐like receptor loci, *lin‐12* and *glp‐1*, which have overlapping but not identical biological roles (Lambie & Kimble 1991). The two loci are the product of a gene duplication event, which has been followed by some degree of subfunctionalization (Rudel & Kimble 2002). We used the new genomic data to study the evolution of these two loci.

**Figure 7:**
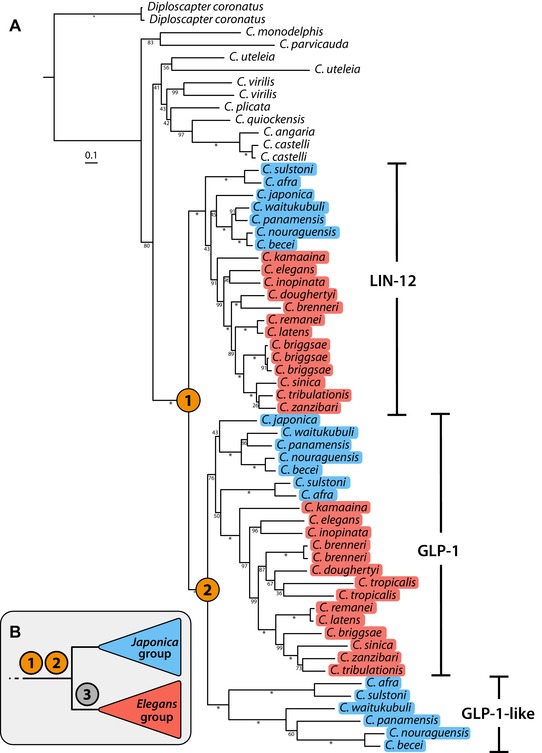
Maximum likelihood gene tree of Notch‐like receptors in *Caenorhabditis*. **(A)** Gene tree of the orthogroup containing *C. elegans* proteins LIN‐12 (CELEG.R107.8) and GLP‐1 (CELEG.F02A9.6) inferred using maximum likelihood (JCCMut+Γ substitution model). *Elegans* and *Japonica* groups are highlighted in red and blue, respectively. Duplication events are denoted by orange circles. Branch lengths represent the number of substitutions per sites; scale is shown. Bootstrap values are displayed as branch annotations, “^*^” = 100. **(B)** Inferred events: (1) Duplication of ancestral Notch‐like receptor gene; (2) Duplication of *glp‐1* gene; (3) Loss of one of the two duplicated *glp‐1* genes in the *Elegans* group.

From an orthology clustering analysis including 27 *Caenorhabditis* species and the outgroup taxon *Diploscapter coronatus*, we identified the orthogroup containing the *C. elegans* proteins LIN‐12 and GLP‐1. We aligned the protein sequences of each member of the orthogroup and reconstructed a phylogenetic tree using maximum likelihood inference. The resulting topology (Fig. 7A) reaffirms that *lin‐12* and *glp‐1* are the product of a gene duplication event, and indicates that this occurred at the base of the *Elegans* supergroup. The genomes of several species outside the *Elegans* supergroup, including the basal taxa *C. monodelphis* and *C. parvicauda*, encode only a single Notch‐like receptor. We also find evidence for a second duplication of *glp‐1* at the base of the *Elegans* supergroup, followed by loss in species belonging to the *Elegans* group, including *C. elegans* (Fig. 7B). Genomes of species belonging to the *Japonica* group have retained both copies of the *glp‐1*‐like gene, and therefore encode three Notch‐like receptor genes in total. Both duplication branches had maximal bootstrap support. Further, within‐species duplications of the Notch‐like receptor genes appear to have occurred in several species, including in *C. uteleia*. In those cases in which the subtending branches are extremely short, such as in *D. coronatus*, these putative within‐species duplications are likely to be an artifact arising from regions of uncollapsed heterozygosity present in the genome assembly.

Both *Elegans* supergroup duplication events were followed by divergence in the rate of substitution of the paralogues, as indicated by branch lengths (Fig. 7A). GLP‐1 and its orthologues underwent increased rates of substitution after the first duplication event relative to LIN‐12 and orthologues, with means of 0.93 and 0.58 substitutions per amino acid site, respectively (Table S7). The paralogues of GLP‐1 found in the *Japonica* group have also undergone increased rates of substitution relative to GLP‐1 after the second duplication event, with means of 1.22 and 0.78 substitutions per site, respectively.

The binding of DSL ligands by Notch receptor proteins is mediated by extracellular epidermal growth factor (EGF)‐like repeats (Rebay et al. 1991). In *C. elegans*, LIN‐12 and GLP‐1 differ in the number of EGF‐like repeats, with 13 and 10, respectively. We counted the number of EGF‐like repeats in all orthologues of LIN‐12 and GLP‐1 using InterProScan and used a stochastic character mapping approach to infer the structure of the ancestral *Caenorhabditis* Notch‐like receptor. We infer that the ancestral receptor possessed 13 EGF‐like repeats and was thus similar in structure to LIN‐12 and its orthologues and to the single Notch‐like receptors of those species that do not belong to the *Elegans* supergroup (Fig. S12; Table S8). We also infer that the loss of three EGF‐like repeats in GLP‐1 and its orthologues occurred prior to the divergence of the *Elegans* supergroup and to the second duplication event (Fig. S12).

## Discussion

### PHYLOGENETIC RELATIONSHIPS IN THE GENUS *CAENORHABDITIS*

Using genomic and transcriptomic data from 32 species of *Caenorhabditis* and the outgroup taxon *D. coronatus*, we have generated the most comprehensive *Caenorhabditis* phylogenetic tree published to date, in terms of number of taxa and number of orthologues sampled. The majority of relationships were recovered with maximal support by all analyses and are consistent with previous studies (Kiontke et al. 2011; Slos et al. 2017). The 10 species described here are sampled from all previously defined major clades in the genus, and include the second‐most early diverging *Caenorhabditis, C. parvicauda*.

Our results corroborate the monophyly of the *Japonica* and *Elegans* groups (and therefore the *Elegans* supergroup) as defined in Kiontke et al. (2011). The majority of relationships within these groups are consistent with previous studies (Kiontke et al. 2011; Slos et al. 2017). However, *C. kamaaina* (sp. 15), which in our analyses was recovered as the most early diverging species in the *Elegans* group, has previously been recovered as sister to the *Japonica* group (Kiontke et al. 2011). Our finding that the *C. guadeloupensis* + *C. uteleia* group is sister to the *Elegans* supergroup is consistent with the tree topology in Slos et al. (2017) and provides evidence that the *Drosophilae* supergroup, as recovered by the analysis of Kiontke et al. (2011), is paraphyletic.

Interestingly, relationships at three nodes were inconsistent across analyses. The placement of *C. japonica* as sister to *C. afra* + *C. sulstoni* and the placement of the clade containing *C. vivipara* + *C. virilis* as sister to the *Elegans* supergroup were recovered by ML analysis only. Both of these relationships disagree with all previously published *Caenorhabditis* phylogenetic trees (Kiontke et al. 2011; Slos et al. 2017). The sister relationship of *C. brenneri* and *C. doughertyi*, as recovered by BI and ML, was recovered in the analyses of Kiontke et al. (2011), but not by Slos et al. (2017).

It is not clear why the different approaches generate inconsistent topologies, and whether these inconsistencies are due to conflict in the data or due to low resolution, which result in ambiguous regions of the topology. Concatenated alignments of large numbers of loci, such as the one used in our BI and ML analyses, may contain genes that have different histories due to processes such as incomplete lineage sorting or introgression through hybridization in the early stages of speciation (Maddison & Wiens 1997). Given sufficient conflicting signals, concatenation approaches are known to lead to inaccurate topologies (Kubatko & Degnan 2007). Coalescent‐based summary approaches, such as ASTRAL‐III, are able to accommodate conflicting signals in gene trees arising from incomplete lineage sorting (Mirarab et al. 2014), but may be sensitive to gene tree estimation error (Gatesy & Springer 2014; Roch & Warnow 2015). The different models used in BI and ML analysis (CAT‐GTR and GTR, respectively) are presumably one source of conflict between these two analyses. The CAT‐general‐time reversible model (GTR), unlike the GTR model, is able to accommodate heterogeneity in the amino acid substitution process at different sites in the alignment (Lartillot & Philippe 2004). Further work is required to determine whether accounting for such heterogeneity is necessary for this dataset.

We feel it premature to redefine the groups and supergroups in *Caenorhabditis* until we have a more complete representation of the known species, and particularly of species currently placed in the *Drosophilae* group. Our data could be used to support a broadening of the *Elegans* supergroup to include *C. uteleia* and *C. guadeloupensis*. Alternately, the *Elegans* supergroup could be retained as the clade including the last common ancestor of *C. elegans* and *C. japonica* and their respective groups, but not *C. uteleia* and *C. guadeloupensis*. Many more species outside the *Elegans* and *Japonica* groups are now available in culture for genome sequencing and subsequent placement on the phylogenetic tree (Kiontke et al. 2011; Félix et al. 2014). As sequencing technologies improve, and chromosomal assembly becomes more achievable (Kanzaki et al. 2018), we would also hope to use a chromosomally resolved genomic tree to uncover additional patterns and reveal novel processes in *Caenorhabditis* evolution.

### GENOME SIZE VARIATION IN *CAENORHABDITIS*

By comparing 24 *Caenorhabditis* species, we found evidence for substantial variation in genome size within the genus, with genome sizes ranging from 65 to 140 Mb.

The smallest genome, that of *C. sulstoni*, differed by 35 Mb from that of the reference *C. elegans* because of reduction in protein‐coding gene number, intron size, intergenic distances, and proportion of repetitive DNA. Surprisingly, while we found that introns in *C. sulstoni* appear to have undergone contraction, we did not find evidence for decreased intron count in its small genome. Using PGLS analysis, we found evidence that genome size is significantly positively correlated with protein‐coding gene number and with proportion of repetitive DNA.

Our analysis of genome size variation assumes that we have accurately estimated the span of each genome in our draft assemblies. In *Caenorhabditis*, in which levels of nucleotide diversity are particularly high in outcrossing species (Cutter et al. 2006; Dey et al. 2013), several published draft genomes are known to contain high levels of uncollapsed heterozygosity, and thus have larger‐than‐expected assembly spans (Barrière et al. 2009). Consistent with this, we found that the genomes of *C. brenneri* and *C. japonica* (with assembly spans of 190 and 166 Mb, respectively) contained high numbers of duplicated loci, as did the genomes of *C. angaria, C. waitukubuli*. The genome of the outgroup taxon, *Diploscapter coronatus*, also has elevated duplication rates. *D. coronatus* is a parthenogen, and its assembly represents two copies of the original haploid genome (Hiraki et al. 2017). These species were excluded from our analyses.

Several studies have established a link between reproductive mode and genome size in *Caenorhabditis*, suggesting that hermaphroditic species have smaller genomes due to gene loss (Thomas et al. 2012; Fierst et al. 2015; Yin et al. 2018). *C. briggsae* appears to have undergone extensive genome contraction after the evolution of hermaphroditism compared to its gonochoristic sister *C. nigoni*. This genome contraction in *C. briggsae* is largely the result of loss of genes with male‐biased expression (Yin et al. 2018). In contrast, *C. elegans* does not show a significant reduction in protein‐coding gene number when compared with its outcrossing sister taxon *C. inopinata*. Instead, it appears that a substantial fraction of the 22.1 Mb difference in genome size between these two species is the result of the expansion of transposable elements in the *C. inopinata* genome (Kanzaki et al. 2018).

Given our findings and those of previous studies, genome size variation within *Caenorhabditis* is driven by multiple, interacting, underlying mechanisms. The relative contributions of contrasting molecular processes, such as gene loss versus gene family expansion, or intergenic contraction versus repeat expansion, and their evolutionary drivers, such as a switch in reproductive mode, must differ between different lineages in the genus. A further, more detailed analysis, involving genomes of other species not analyzed here, including that of *C. wallacei*, the outcrossing sister taxon of *C. tropicalis*, will be required before we are able to identify general trends, and specific mechanisms, responsible for genome size variation within the genus.

### THE EVOLUTION OF NOTCH/LIN‐12 SIGNALLING PROTEINS IN *CAENORHABDITIS*

*C. elegans* is a major developmental biology model, and understanding how its particular instance of nematode (and animal) development was established may help reveal the logic of the evolution of development and the importance of processes such as developmental systems drift, wherein similar phenotypes are formed by divergent developmental processes (True & Haag 2001). We studied the evolution of Notch‐like receptor genes in the genomes of 27 species of *Caenorhabditis* and in *D. coronatus*, and found that the *C. elegans* genes *lin‐12* and *glp‐1* are the product of a gene duplication event that occurred at the base of the *Elegans* supergroup. We also found evidence of a subsequent duplication of *glp‐1* on the same branch, followed by loss at the base of the *Elegans* group. The genomes of *Japonica* group species have three Notch‐like receptor genes. We found that *lin‐12* and its orthologues were more conserved, with a lower rate of substitution compared to *glp‐1* and its orthologues and an EGF‐like repeat structure that is more similar to that of the inferred ancestral Notch‐like receptor.

Previous genetic analyses of *lin‐12* and *glp‐1* identified several shared and receptor‐specific developmental functions, suggesting subdivision of ancestral roles after duplication (Lambie & Kimble 1991). While LIN‐12 and GLP‐1 proteins have diverged in sequence, it is likely that a substantial amount of this divergence in function is due to differences in expression patterns of these two proteins as GLP‐1 is capable of performing LIN‐12‐specific roles when placed under the control of *lin‐12* regulatory sequences (Fitzgerald et al. 1993). The extent to which ancestral roles have been subdivided (subfunctionalization), or whether novel roles have been acquired (neofunctionalization), and to what extent these changes are due to changes in expression pattern, could be addressed by studying the function of the ancestral Notch‐like receptor gene in species outside the *Elegans* supergroup. Particularly exciting are the third Notch‐like receptor genes present in the genome of *Japonica* group species that appear to have undergone a substantial increase in substitution relative to *glp‐1* and its orthologues after duplication. Genetic analyses of Notch‐like receptors, which have yet to be carried out in these species, could reveal the implications of this increased rate of substitution, including whether further subdivision of ancestral roles has occurred or novel roles have been acquired.

### GENOMICS, SPECIES DESCRIPTION, AND *CAENORHABDITIS* BIOLOGY

By describing new species of *Caenorhabditis* with high‐quality genomic data we hope to promote not only understanding of the evolution of this exciting genus, but also exploitation of these new species in deepening understanding of the pattern and process of organismal evolution. In particular, the ability to interfere with gene function through RNAi and accurately modify genomes using CRISPR‐Cas editing is routine in *C. elegans* and becoming established in other species. Using the genomic data presented here, specific loci can be deleted or altered to test hypotheses of gene function in a rich phylogenetic context.

Currently, strains corresponding to about 50 species of *Caenorhabditis* are available in culture. Our goal is to provide genome sequences and formal species descriptions for all these species. The assemblies presented here are not chromosomal, unlike the genome of *C. elegans*, because of the inability of short‐read data to bridge and resolve large repeats. Recent developments in genomic technologies make phased, chromosomal assemblies of all *Caenorhabditis* species’ genomes an achievable goal, and we and others are using long read and other approaches to advance this goal.

Approximately 20,000 new species are described each year. We also suggest that, wherever technically possible, the generation of genomic data as part of the introduction of new taxa should become standard. Complete sequencing of all taxa on earth has been proposed (Lewin et al. 2018), and active support should be offered to taxonomists to add their new species to this global effort.

## Methods

### SAMPLING AND ISOLATION

See Kiontke et al. (2011) and (Barrière & Félix 2014) for details of sampling strategies. Briefly, rotting vegetable matter samples were collected and stored in plastic bags. The samples were then analyzed in the laboratory by placing them onto 90 mm NGM agar plates (Stiernagle 2006), seeded in the center with *Escherichia coli* OP50. *Caenorhabditis* species are generally attracted by the *E. coli* lawn. Isofemale lines were established by isolation of a single female that was already mated or by co‐culturing one female and one male. A list of isolates is available in Table S1.

### CULTURE AND FREEZING

Nematodes were cultured on NGM agar plates and frozen with standard *C. elegans* protocols (Stiernagle 2006). Some species, such as *C. parvicauda* do not grow well on *E. coli* OP50 and are better kept with some of their original microbial environment. Like other species in the *Angaria* group, *C. quiockensis* does not survive well freezing and thawing with the standard *C. elegans* protocol and was heat‐shocked for 1–2 hrs at 37°C before freezing.

### CROSSES AND ASSIGNMENT OF ISOLATES TO SPECIES

For each of the interspecific crosses with results presented in Table S2, five L4 females and five L4 or adult males were placed together on a 55 mm agar plate seeded with *E. coli* OP50. The plate was checked regularly for presence and cross‐fertility of the progeny. To assign additional isolates (listed in Table S1), either crosses were performed, or barcode sequence alone was considered sufficient if it was identical to the barcode of a reference strain.

### INBREEDING AND NUCLEIC ACID PREPARATION

For inbreeding, one L4 female and one male were used to seed each generation. Inbreeding was performed for 25–26 generations.

After thawing, each inbred strain was bleached and grown on 90 mm NGM plates enriched with agarose (for 1 L: 3 g NaCl, 5 g bacto‐peptone, 10 g agar, 7 g agarose, 1 mL cholesterol 5 mg/mL in ethanol, 1 mL CaCl_2_ 1 M, 1 mL MgSO_4_ 1 M, 25 mL KPO_4_ 1 M). Worms were harvested just after starvation and washed in M9 several times to remove *E. coli*.

For genomic DNA extraction, the nematode pellets were resuspended in 600 μL of Cell Lysis Solution (Qiagen) complemented with 5 μL of proteinase K (20 μg/μL) and incubated overnight at 56°C with shaking. The day after, the lysates were incubated for one hour at 37°C with 10 μL of RNAse A (20 μg/μL) and the proteins were precipitated with 200 μL of protein precipitation solution (Qiagen). After centrifugation, the supernatants were collected in new tubes and genomic DNA were precipitated with 600 μL of isopropanol. The pellets were washed in ethanol 70% and dried one hour before being resuspended in 50 μL of DNAse free‐water.

For RNA extraction, 100 μL of nematode pellet was resuspended in 500 μL of Trizol (5 volumes of Trizol per volume of pelleted nematodes). The Trizol suspension was frozen in liquid nitrogen and then transferred to a 37°C water bath to be thawed completely. This freezing/thawing process was repeated four to five times and the suspension was vortexed for 30 sec and let rest for 30 sec (five cycles). A total of 100 μL chloroform was added and the tubes were shaken vigorously by hand for 15 sec and incubated for 2–3 min at room temperature. After centrifugation (15 min at 13,000 rpm and 4°C), the aqueous (upper) phase containing the RNA was transferred to a new tube and precipitated with 250 μL of isopropanol. The pellets were washed in 70% ethanol and dried 15–20 min before being resuspended with 50–100 μL of RNAse‐free water. An aliquot of each DNA and RNA preparation was run on agarose gel to check their quality and quantitated with Qubit (Thermo Scientific)

### GENOME SEQUENCING

For *C. parvicauda, C. quiockensis, C. sulstoni, C. tribulationis, C. uteleia, C. waitukubuli*, and *C. zanzibari*, two short‐insert (insert sizes of 300 and 600 bp, respectively) genomic libraries and a single short‐insert (150 bp) RNA library were prepared using Illumina Nextera reagents and sequenced (125 bases, paired‐end) on an Illumina HiSeq 2500 at Edinburgh Genomics (Edinburgh, UK). For *C. becei and C. panamensis*, a short‐insert (insert size of 600 bp) genomic library and a long‐insert (insert size of 4 kb) genomic library were prepared using Illumina Nextera reagents and sequenced (100 bases, paired‐end) on an Illumina HiSeq 2500 at the NYU Center for Genomics and Systems Biology GenCore facility (New York, USA). All raw data have been deposited in the relevant International Nucleotide Sequence Database Collaboration databases (Table 1).

### *DE NOVO* GENOME ASSEMBLY AND GENE PREDICTION

Detailed methods for each species, along with all software tools used (including versioning and command line options), are available in Supporting Information 2. We performed quality control of all raw sequence data using FastQC (Andrews 2010) and used Skewer (Jiang et al. 2014) and FASTQX Toolkit (Gordon & Hannon 2010) to remove low‐quality bases and adapter sequence. Adapters were removed from long‐insert data using NextClip (Leggett et al. 2014). For each species, we identified contaminants using taxon‐annotated, GC‐coverage plots as implemented in blobtools (Laetsch & Blaxter 2017), preliminary assemblies were generated using CLC Bio (CLCBio, Copenhagen, Denmark), and likely taxon origin determined using NCBI‐BLAST+ (Camacho et al. 2009) or using Kraken (Wood & Salzberg 2014). Reads originating from contaminant genomes were discarded. We estimated the optimal k‐mer length for assembly independently for each genome using KmerGenie (Chikhi & Medvedev 2014). Preliminary assemblies were generated using several de Bruijn graph assemblers, including Velvet (Zerbino & Birney 2008) and Platanus (Kajitani et al. 2014), across several parameter values. The resulting assemblies were assessed using numerical metrics, and two biological completeness metrics, CEGMA (Parra et al. 2007), and BUSCOs (Simão et al. 2015; using the ‘Nematoda_ob9’ dataset). For each species, the highest quality assembly was selected and, where possible, scaffolded with assembled transcripts using SCUBAT2 (https://github.com/GDKO/SCUBAT2) or with long‐insert “mate‐pair” data using Platanus.

We identified repeats independently in each genome using RepeatModeler (Smit & Hubley 2010). After filtering sequences that likely originated from protein‐coding genes, we combined each repeat library with known Rhabditida repeats obtained from RepBase (Jurka et al. 2005). This concatenated repeat library was then provided to RepeatMasker (Smit et al. 1996) for masking. If RNA‐seq data were available, reads were aligned to the assembly using STAR (Dobin et al. 2013) and the resulting BAM file provided to BRAKER (Hoff et al. 2016), which performed final gene prediction. If RNA‐seq data were not available, genes were predicted initially using MAKER2 (Hoff et al. 2016) with a training set composed of ab initio predictions from GeneMark (Lukashin & Borodovsky 1998), gene models identified by CEGMA (Parra et al. 2007), and the *C. elegans* protein sequence set. The resulting gene models were used to train AUGUSTUS (Keller et al. 2011), which generated the final gene set.

### PHYLOGENETIC ANALYSIS

Accessions to all data used in phylogenomics analysis are available in Table S9. For those species with available genome sequences, we identified and collected the protein sequence of the longest isoform of each protein‐coding gene. For those species for which only transcriptome data was available (*C. guadeloupensis* and *C. vivipara*), open reading frames and putative protein sequences were predicted using TransDecoder (Haas & Papanicolaou 2012). OrthoFinder (Emms & Kelly 2015; using the default inflation value of 1.5) was used to cluster all protein sequences into putatively orthologous groups (OGs). OGs containing loci present as single copy in at least 27 of the 33 species (except in those species with genome assemblies known to contain regions of uncollapsed heterozygosity [i.e., *C. angaria*, *C. brenneri*, *C. japonica*, *C. waitukubuli*, and *D. coronatus*] and in those species for which only transcriptome data were available, where counts of two were allowed) were selected.

To identify paralogous sequences, we aligned the protein sequences of each OG using FSA (Bradley et al. 2009) and generated a maximum likelihood tree along with 100 rapid bootstraps using RAxML (Stamatakis 2014)(using best‐fitting amino acid substitution, as selected by the model testing component of RAxML, and gamma‐distributed rate variation among sites). Each tree was screened by PhyloTreePruner (Kocot et al. 2013; collapsing nodes with bootstrap support <50), and any OGs containing paralogues were discarded. If two representative sequences were present for any species (i.e., “in‐paralogues”) after this paralogue screening step, the longest of the two sequences was retained and the other discarded.

The protein sequences of each one‐to‐one OG were then aligned using FSA and gene trees estimated as previously described. ASTRAL‐III (Mirarab & Warnow 2015), a coalescent‐based method, was then used to reconstruct the species phylogeny using individual genes trees as an input. We also reconstructed the species tree using a concatenation approach. TrimAl (Capella‐Gutiérrez et al. 2009) was used to remove spuriously aligned regions from each alignment, which were subsequently concatenated using catfasta2phyml (available at https://github.com/nylander/catfasta2phyml). ML analysis was performed with RAxML (general‐time reversible model (GTR) (Tavaré 1986) with gamma‐distributed rate variation among sites) along with 100 bootstrap replicates. Bayesian inference was carried out using the site‐heterogeneous CAT‐GTR substitution model (Lartillot & Philippe 2004) (with gamma‐distributed rate‐variation among sites) implemented in PhyloBayes MPI (Lartillot et al. 2013), with two independent chains. Convergence was assessed using Tracer (Rambaut et al. 2007). A posterior consensus tree was estimated using samples from both chains, with the initial 10% of all trees discarded as burn‐in. Newick trees were visualized using the iTOL web server (Letunic & Bork 2016).

### MORPHOLOGICAL CHARACTER MAPPING

Morphological characters and phenotypes were encoded as “1” or “0” depending on their presence or absence (Table S4). Ancestral morphological character states were inferred using the stochastic mapping implemented in the phytools package (Revell 2012). Using the equal rates model of evolution, we simulated 1000 character histories on the species tree in Fig. 1A and summarized the character histories as posterior probabilities on internal nodes.

### GENOME SIZE ANALYSIS

For those species for which gene structure information was available, we collected the longest isoform of each protein‐coding gene. As previously described, we clustered protein sequences into putatively orthologous groups using OrthoFinder. To identify genome assemblies that might contain uncollapsed haploid segments and thus expanded genome spans, we selected orthogroups containing loci that were present at least 22 of the 28 species and that were on average single copy. For each species, we counted the number of loci present in the selected orthogroups, and divided this count by the total number of orthogroups that contained a representative sequence for that species. We excluded genome assemblies that had a duplication ratio of >1. 2 (*C. angaria*, *C. brenneri*, *C. japonica*, *C. waitukubuli*, and *Diploscapter coronatus*) from downstream analyses of genome size (Table S10). We collected summary statistics for each genome assembly using custom scripts and performed PGLS analyses using the ape R package in (Paradis & Schliep 2018), using the Brownian model of evolution and the phylogenetic tree in Figure 1A.

### NOTCH‐LIKE RECEPTOR ANALYSIS

In an existing orthology clustering set, we identified the OG that contained the *C. elegans* proteins LIN‐12 (R107.8) and GLP‐1 (F02A9.6) and collected the protein sequences of each member. After removing sequences that were shorter than 700 amino acids, we generated an amino acid alignment using FSA. We performed a maximum likelihood analysis using RAxML, allowing the substitution model to be automatically selected, and conducted 100 rapid bootstrap replicates. Branch lengths were extracted using a custom Python script (available at https://github.com/lstevens17/caeno-ten-descriptions), making use of the ete3 module (Huerta‐Cepas et al. 2016). We identified conserved domains in each protein sequence using InterProScan (Jones et al. 2014). Counts of EGF‐like repeats were obtained from the ProSiteProfiles database (release 2017_09; Sigrist et al. 2013). To infer the ancestral EGF‐like repeat count, we used the stochastic mapping approach implemented in the phytools package (Revell 2012). Using the equal rates model of evolution, we simulated 1000 character histories on the gene tree inferred by RAxML, and summarized the character histories as posterior probabilities on internal nodes.

### SCANNING ELECTRON MICROSCOPY

Nematode cultures were resuspended and washed twice in M9 solution, then fixed overnight at 4°C in M9 or 50 mM phosphate pH 7.0 + glutaraldehyde 2.5 to 4%, depending on the batch. The fixed animals were rinsed twice in M9 and dehydrated through an ethanol series, pelleting them at each step at 1 g in a tube. The samples were processed through critical point drying and coating with 20 nm of Au/Pd, and then observed with a JEOL 6700F microscope at the Ultrastructural Microscopy Platform of the Pasteur Institute.

### NOMARSKI MICROGRAPHS

The Nomarski micrographs were taken using an AxioImager 2 (Zeiss) after mounting the animals on a Noble agar pad as described in (Shaham 2006). The pictures showing extruded spicules were taken after exposing the animals for 2 sec in the microwave before adding the coverslip.

Associate Editor: S. Wright

## Supporting information


**Table S1**. List of isolates and their origin.
**Table S2. Mating tests**. This table contains several sheets, showing the results of crosses between isolates of different species. Successful crosses are labeled in green.“100s of embryos” refer to unhatched dead embryos remaining on the plate.
**Table S3**. Detailed genome assembly and gene prediction statistics.
**Table S4. Morphological characters used for ancestral state reconstruction**. ‘1’ denotes presence or existence; ‘0’ denotes absence.
**Table S5. Genome contents of *C. sulstoni* and *C. elegans***. Gene structure statistics were calculated using the longest isoform of each protein‐coding gene. UTR regions were not annotated in *C. sulstoni* and so were not considered in either species.
**Table S6. Genome statistics used in PGLS analysis**. Gene structure statistics were calculated using the longest isoform of each protein‐coding gene. UTR regions were not considered as they were not annotated in several species. Repeat content was estimated *de novo* using RepeatModeler and RepeatMasker.
**Table S7. Mean branch lengths from Maximum likelihood gene tree of all Notch‐like proteins**. Branch lengths were extracted using a custom Python script (available at https://github.com/lstevens17/caeno-ten-descriptions).
**Table S8. EGF‐like repeat counts for LIN‐12/GLP‐1 homologues**. Counts of EGF‐like repeats were obtained from were obtained from the ProSiteProfiles database (release 2017_09).
**Table S9. Accessions and links to data used in phylogenomic analysis**.
**Table S10. Completeness and duplication statistics for 28 *Caenorhabditis* species based on 8,286 orthologues**. We selected groups of orthologues which were present in at least 22 species and had a mean count of 1. The duplication ratio was calculated by dividing the total number of sequences present for each species by the total number of orthogroups which contained a representative sequence for that species.
**Figure S1. Assembly spans and genome size estimates**. Kmers of length 19 were counted using KMC (v2.3). Genome size was estimated using GenomeScope (Vurture et al. 2017). The model used by GenomeScope to estimate genome size did not converge for *C. waitukubuli* (presumably due to high heterozygosity) and is not shown.
**Figure S2. Kmer spectra for *C. waitukubuli* (sp. 39)**. Kmers were counted using KMC (v2.3). Plotted in R using the ggplot2 package.
**Figure S3. PhyloBayes phylogenetic tree**. Phylogenetic tree inferred using Bayesian inference with the CAT‐GTR+Γ substitution model. Bayesian posterior probabilities are 1.0 unless noted as branch annotations. Scale is in substitutions per site.
**Figure S4. RAxML phylogenetic tree**. Maximum likelihood phylogenetic inferred using RAxML with the GTR+Γ substitution model. Bootstrap support values (100 replicates) are 100 unless noted as branch annotations. Scale in substitutions per site.
**Figure S5. ASTRAL‐III phylogenetic tree**. Phylogenetic tree inferred using ASTRAL‐III, by providing maximum likelihood gene trees (inferred using RAxML with the substitution model selected automatically) as input. As ASTRAL‐III outputs trees with branch lengths in coalescent units, branch lengths in substitutions per site were estimated using RAxML with the GTR+Γ substitution model and the concatenated alignment. Bayesian posterior probabilities are 1.0 unless noted as branch annotations. Scale is in substitutions per site.
**Figure S6. Morphology of *C. parvicauda* male tail. A, A’**: Two focal planes in Nomarski optics of the same adult male tail of strain NIC134. **B**: Scanning electron microscopy, strain JU2070. ad: anterior dorsal papilla; pd: posterior dorsal papilla; vr1, etc.: ventral papilla 1, etc. These photographs exemplify the left‐right asymmetry of the posterior papillae, observed in strains NIC134 and JU2070, as well as JU1766 (not shown).
**Figure S7. Morphology of *C. quiockensis* and *C. vivipara*. A‐C**: Nomarski micrographs of male tails of *C. quiockensis* strain JU2745. **D**: *C. castelli* JU1426 is shown for comparison. Same scale in A‐D. **E**: *C. vivipara* NIC1070 male tail **F‐H**: Details of pericloacal regions of other animals. Note the much larger and wider fan in *C. vivipara*. Ad: anterior dorsal papilla; gub: gubernaculum; pd: posterior dorsal papilla; spic: spicule; v1, etc.: ventral papillae.(A,B,D, H) are lateral views. (C) and (E‐G) are ventral views. **I**: Gravid female adult with late‐stage embryos in the uterus and a recently laid L1 stage larva. Bars: 10 μm, except 5 μm for (F,G) and 50 μm for (I). Anterior is to the left except in H where the anterior is to the top.
**Figure S8. Male tails of species of the *Elegans* supergroup**. Ventral views by Nomarski optics of *C. becei* strain QG704, *C. waitukubuli* strain NIC564, *C. panamensis* strain QG702, *C. sulstoni* strain SB454 and *C. afra* JU1199 male tails. Note the variation in the respective lengths of rays 4 and 5. All pictures at are at the same scale. Bar: 10 μm. The correspondence between the v1‐7, ad, pd papilla nomenclature and that of rays used in *C. elegans* is indicated on the *C. becei* picture. v1‐7 denote ventral papillae, while ad and pd denote the anterior and posterior dorsal papillae, respectively. In the *C. elegans* nomenclature, rays are instead numbered r1‐r9 without distinction between ventral and dorsal rays.
**Figure S9. Ancestral state reconstruction of male tail characters**. **A**: Antero‐posterior position of the dorsal ray. **B**: Closed fan. Ancestral state reconstruction was performed by generating 1000 stochastic character maps of each morphological character on the phylogenetic tree in Fig. 1A using the equal rates model of evolution. Pie charts on internal nodes represent poster probabilities of ancestral states. The fan of *C. vivipara* was ambiguous and thus excluded from the open fan character mapping analysis.
**Figure S10. Spicule tip shape in *Elegans* supergroup species**. Nomarski micrographs. Left column: ventral view. Right column: ventro‐lateral view. The tip of spicules in *C. afra* and *C. sulstoni* (as in several species of the *Japonica* group; Kiontke et al. 2011) is broad and bent with a discontinuity on the curvature, compared to a thin and continuously bent tip in species of the *Elegans* group such as *C. zanzibari* or *C. elegans*. That of *C. panamensis* strain QG702 is quite thin, while that of *C. becei* strain QG704 is broad. Bar for all panels: 5 μm.
**Figure S11. Phylogenetic generalized least squares (PGLS) analysis of genome size and contents**.
**A**: Protein‐coding gene number. **B**: Estimated repeat content. **C**: Mean intron count per gene. **D**: Mean intron size. **E**: Mean intergenic distance. Phylogenetic tree presented in Fig. 1A was used in all analyses; points are coloured based on major clades. Gene structure statistics were calculated using the longest isoform of each protein‐coding gene. UTR regions were not considered as they were not annotated in several species. Repeat content was estimated *de novo* using RepeatModeler and RepeatMasker.
**Figure S12. Ancestral state reconstruction of EGF‐like repeat structure**. Ancestral state reconstruction was performed by generating 1000 stochastic character maps of EGF‐like repeat count on the gene tree presented in Fig. 7A using the equal rates model of evolution. Pie charts on internal nodes represent poster probabilities of ancestral states.
**Document S1. Species declarations**.
**Document S2. Detailed bioinformatics methods**. Methods, versions and relevant parameters used in genome assembly, gene prediction, phylogenomic analysis, genome size analysis, and Notch‐receptor analysis.
