# Supplementary material for: Comparative genomics of 10 new Caenorhabditis species
Source: Evol Lett. 2019 Apr 2;3(2):217–36. doi: 10.1002/evl3.110 (PMC6457397; doi:10.1002/evl3.110)
Supplement: Supplementary file 1 — Table S1. List of isolates and their origin. Table S2. Mating tests. This table contains several sheets, showing the results of crosses between isolates of different species. Successful crosses are labeled in green.“100s of embryos” refer to unhatched dead embryos remaining on the plate. Table S3. Detailed genome assembly and gene prediction statistics. Table S4. Morphological characters used for ancestral state reconstruction. ‘1’ denotes presence or existence; ‘0’ denotes absence. Table S5. Genome contents of C. sulstoni and C. elegans. Gene structure statistics were calculated using the longest isoform of each protein‐coding gene. UTR regions were not annotated in C. sulstoni and so were not considered in either species. Table S6. Genome statistics used in PGLS analysis. Gene structure statistics were calculated using the longest isoform of each protein‐coding gene. UTR regions were not considered as they were not annotated in several species. Repeat content was estimated de novo using RepeatModeler and RepeatMasker. Table S7. Mean branch lengths from Maximum likelihood gene tree of all Notch‐like proteins. Branch lengths were extracted using a custom Python script (available at https://github.com/lstevens17/caeno-ten-descriptions). Table S8. EGF‐like repeat counts for LIN‐12/GLP‐1 homologues. Counts of EGF‐like repeats were obtained from were obtained from the ProSiteProfiles database (release 2017_09). Table S9. Accessions and links to data used in phylogenomic analysis. Table S10. Completeness and duplication statistics for 28 Caenorhabditis species based on 8,286 orthologues. We selected groups of orthologues which were present in at least 22 species and had a mean count of 1. The duplication ratio was calculated by dividing the total number of sequences present for each species by the total number of orthogroups which contained a representative sequence for that species. Figure S1. Assembly spans and genome size estimates. Kmers of length 19 were counted usi [file EVL3-3-217-s001.zip › evl3110-sup-0001-SuppMat/evl3110-sup-0001-SuppMat.docx]

Supplementary File 1 - Species Declarations

The electronic edition of this article conforms to the requirements of the amended International Code of Zoological Nomenclature, and hence the new names contained herein are available under that Code from the electronic edition of this article. This published work and the nomenclatural acts it contains have been registered in ZooBank, the online registration system for the ICZN. The ZooBank LSIDs (Life Science Identifiers) can be resolved and the associated information viewed through any standard web browser by appending the LSID to the prefix ‘‘http://zoobank.org/’’. The LSID for this publication is: urn:lsid:zoobank.org:pub:59A27812-4C47-42CE-AF4B-A2E41DBD8A26. The electronic edition of this work was published in a journal with an ISSN. A RhabditinaDB database with the species taxonomic status and morphology is available at [rhabditina.org](http://rhabditina.org)

### *Caenorhabditis parvicauda* Stevens and Félix sp. n.

Zoobank identifier urn:lsid:zoobank.org:act:2EBBCC53-8388-40CC-B7D4-D921BB07353E

= *Caenorhabditis* sp. 21 in [Slos *et al.* (2017)](https://paperpile.com/c/f6CXok/OzZ1t)

The type isolate by present designation is NIC206, deposited at the Caenorhabditis Genetics Center. The species is delineated and diagnosed by the fertile cross with the type isolate NIC206 in both cross directions, yielding highly fertile hybrid females and males that are interfertile and cross-fertile with their parent strains. The species reproduces through males and females. This species differs by SSU, LSU and ITS2 DNA sequences from all species listed in Tables 1 and 2 of [Félix *et al.* (2014)](https://paperpile.com/c/f6CXok/yclVa), *C. sinica* [(Huang *et al.* 2014)](https://paperpile.com/c/f6CXok/hGWP7), *C. monodelphis* [(Slos *et al.* 2017)](https://paperpile.com/c/f6CXok/OzZ1t), *C. dolen*s, *C. astrocarya* [(Ferrari *et al.* 2017)](https://paperpile.com/c/f6CXok/zqaoI) and Table 1 of the present article. Note that these ribosomal DNA sequences may vary within the species. The type isolate was collected from a rotting fruit collected on the island of Sainte-Marie, Madagascar in December 2010. The mouth is endowed with the usual set of sensory organs disposed in a concentric manner, namely six labial sensillae, two amphids and six male-specific cephalic sensillae (Fig. 1A). Three ridges can be seen in the lateral field of adults of both sexes (Fig. 1C,D). The adult female tail end is long and thin (Fig. 1E). The male tail displays no fan, and short papillae (Fig. 1F, I and S5). The antero-posterior positions of the anterior dorsal papilla (ad) and the most posterior ventral papilla v6-7 appear asymmetric between the left and right sides of the body. The spicules are thick, with a complex tip (Fig. 1G). Simple pre- and post-cloacal sensilla can be seen in Fig. 1H. The males mate in a spiral position. The species is named after its narrow male tail without an extended fan.

#### Note on *C. parvicauda*

The species does not grow on *E. coli* OP50 alone (where it develops into developmentally arrested dauer larvae), and requires associated bacteria. NIC206 (reference strain) is a culture derivative of NIC134 that grows better, while NIC534 is a 25x inbred derivative of NIC134. A first isolate with a similar morphology, JU1766, was collected from a rotting mango collected in August 2009 by Kevin Howan in Faa’a, Tahiti, and subsequently lost. Another isolate, JU2070, was isolated in April 2011 from a rotting fig sampled by Howard Baylis next to a waterfall in Khao Sok National Park, Thailand. Note that the cross between NIC134 (the ancestor of NIC206) and JU2070 tended to produce dauer larvae and a subset of adult females displayed an uncoordinated locomotion. As the *E. coli* culture plates are not ideal for these isolates, we did not raise a new species because of this developmental arrest, although more work on these isolates may suggest to do so. Two isolates with a similar ITS2 (JU2769 and JU2770) were subsequently isolated in 2014 from rotting fruits sampled by Sarah Mühlberger close to Saint-François in Martinique. Thus, this species or species complex appears to be spread around the globe in equatorial regions.

### *Caenorhabditis zanzibari* Stevens and Félix sp. n.

Zoobank identifier

urn:lsid:zoobank.org:act:E3F16CAC-3EEE-4A91-A9BB-B58E6537130E

= *Caenorhabditis* sp. 26 in [Slos *et al.* (2017)](https://paperpile.com/c/f6CXok/OzZ1t)

The type isolate by present designation is JU2161, deposited at the *Caenorhabditis* Genetics Center. The species reproduces through females and males. The species is delineated and diagnosed by the fertile cross with the type isolate JU2161 in both cross directions, yielding highly fertile hybrid females and males that are interfertile and cross-fertile with their parent strains. This species differs by SSU, LSU and ITS2 DNA sequences from all species listed in Tables 1 and 2 of Félix *et al.* (2014), *C. sinica* [(Huang *et al.* 2014)](https://paperpile.com/c/f6CXok/hGWP7), *C. monodelphis* [(Slos *et al.* 2017)](https://paperpile.com/c/f6CXok/OzZ1t), *C. dolen*s, *C. astrocarya* [(Ferrari *et al.* 2017)](https://paperpile.com/c/f6CXok/zqaoI) and Table 1 of the present article. Note that these ribosomal DNA sequences may vary within the species. From molecular data, the closest species are *C. sinica* and *C. tribulationis*, with which it does not form any adult progeny (Table S2). The type isolate was collected from a rotting mandarin fruit collected in the Dole district, Zanzibar, Tanzania (GPS -6.1074, 39.2515) on 7 March 2012. Other isolates were found in Zanzibar, Madagascar, and Mayotte. See Fig. 4A,E,G and Fig. S7 for pictures of male genitalia. The fan is wide, closed anteriorly and the dorsal rays are in antero-posterior positions 5 and 7. The anterior side of the hook shows a distinctive three-lobed shape that is shared with *C. sinica* [(Huang *et al.* 2014)](https://paperpile.com/c/f6CXok/hGWP7) and *C*. *tribulationis*. The males mate in a parallel position. The species is named after its place of isolation.

### *Caenorhabditis panamensis* Stevens and Félix sp. n.

Zoobank identifier

urn:lsid:zoobank.org:act:EE353270-9570-483A-9058-DFAED3A12A93

= *Caenorhabditis* sp. 28

The type isolate by present designation is QG702, deposited at the *Caenorhabditis* Genetics Center. The species reproduces through females and males. The species is delineated and diagnosed by the fertile cross with the type isolate JU702 in both cross directions, yielding highly fertile hybrid females and males that are interfertile and cross-fertile with their parent strains. This species differs by SSU, LSU and ITS2 DNA sequences from all species listed in Tables 1 and 2 of Félix *et al.* (2014), *C. sinica* [(Huang *et al.* 2014)](https://paperpile.com/c/f6CXok/hGWP7), *C. monodelphis* [(Slos *et al.* 2017)](https://paperpile.com/c/f6CXok/OzZ1t), *C. dolen*s, *C. astrocarya* [(Ferrari *et al.* 2017)](https://paperpile.com/c/f6CXok/zqaoI) and Table 1 of the present article. Note that these ribosomal DNA sequences may vary within the species. From molecular data, the closest species are *C. nouraguensis, C. yunquensis,* *C. macrosperma, C. waitukubuli*, and *C. becei*, with which it does not form any larval or adult progeny (Table S2). The type isolate was collected from a rotting palm fruit collected on Barro Colorado Island, Panama (GPS 9.16093, -79.84150) on 24 April 2012 by M. Rockman. A total of 16 isofemale lines were founded from worms collected from 8 different samples on Barro Colorado Island, primarily rotting flowers of *Gustavia superba*. See Fig. S6 and Fig. S7 for pictures of male genitalia. The fan is wide, closed anteriorly and the dorsal rays are in antero-posterior positions 5 and 7. Ray 4 is short in the reference strain (Fig. S6). The males mate in a parallel position. The species is named after the collection locality.

### *Caenorhabditis becei* Stevens and Félix sp. n.

Zoobank identifier

urn:lsid:zoobank.org:act:E0A77D8A-0054-46E5-8558-339F5DA59A4C

= *Caenorhabditis* sp. 29

The type isolate by present designation is QG704, deposited at the *Caenorhabditis* Genetics Center. The species reproduces through females and males. The species is delineated and diagnosed by the fertile cross with the type isolate JU704 in both cross directions, yielding highly fertile hybrid females and males that are interfertile and cross-fertile with their parent strains. This species differs by SSU, LSU and ITS2 DNA sequences from all species listed in Tables 1 and 2 of Félix *et al.* (2014), *C. sinica* [(Huang *et al.* 2014)](https://paperpile.com/c/f6CXok/hGWP7), *C. monodelphis* [(Huang *et al.* 2014)](https://paperpile.com/c/f6CXok/hGWP7), *C. dolen*s, *C. astrocarya* [(Ferrari *et al. 2017)*](https://paperpile.com/c/f6CXok/zqaoI) and Table 1 of the present article. Note that these ribosomal DNA sequences may vary within the species. From molecular data, the closest species are *C. nouraguensis, C. yunquensis,* *C. macrosperma, C. waitukubuli*, and *C. panamensis* with which it does not form any larval or adult progeny (Table S2). The type isolate was collected from a rotting flower of *Gustavia superba* collected on Barro Colorado Island, Panama (GPS 9.15370, -79.82607) on 25 April 2012. A total of 30 isofemale lines of this species were founded from worms collected from 13 different samples of rotting fruit and flowers on Barro Colorado Island. See Fig. 6 and Fig. S7 for pictures of male genitalia. The fan is wide, closed anteriorly and the dorsal rays are in antero-posterior positions 5 and 7. Ray 4 is short in the reference strain (Fig. S6). The males mate in a parallel position. The species is named after the collection locality.

### *Caenorhabditis uteleia* Stevens and Félix sp. n.

Zoobank identifier

urn:lsid:zoobank.org:act:88B1549B-DE53-4A24-AAC3-DD47AE8181B6

= *Caenorhabditis* sp. 31 in [Slos *et al.* (2017)](https://paperpile.com/c/f6CXok/OzZ1t)

The type isolate by present designation is JU2469, deposited at the *Caenorhabditis* Genetics Center. The species reproduces through females and males. The species is delineated and diagnosed by the fertile cross with the type isolate JU2469 in both cross directions, yielding highly fertile hybrid females and males that are interfertile and cross-fertile with their parent strains. The species reproduces through males and females. This species differs by SSU, LSU and ITS2 DNA sequences from all species listed in Tables 1 and 2 of Félix *et al.* (2014), *C. sinica* [(Huang *et al.* 2014)](https://paperpile.com/c/f6CXok/hGWP7), *C. monodelphis* [(Slos *et al.* 2017)](https://paperpile.com/c/f6CXok/OzZ1t), *C. dolen*s, *C. astrocarya* [(Ferrari *et al.* 2017)](https://paperpile.com/c/f6CXok/zqaoI) and Table 1 of the present article. Note that these ribosomal DNA sequences may vary within the species. The type isolate was collected from a rotting fruit collected in Madre de Dios, Peru (GPS -12.801653, -69.289488) on 9 Jan 2013. A second independent isolate (JU2458) was found in the same location. See Fig. 2 for pictures of mouth, lateral ridges and male genitalia. The male tail fan shows a distinctive complex shape at its posterior margin, with one large central valley and two smaller ones on the left and right sides (Fig. 2C,D). The fan is opened anteriorly. The dorsal rays are in antero-posterior positions 5 and 7. The pre-cloacal sensilla is on a hook, itself in between two characteristic lateral folds. The spicule tip is pointed. The post-cloacal sensillae can be seen in Fig. 2C’,D’,F. The species is named after the U-shape of its male tail end.

### *Caenorhabditis sulstoni* Stevens and Félix sp. n.

Zoobank identifier

urn:lsid:zoobank.org:act:D223350B-13F5-4384-A7C4-57844364F676

= *Caenorhabditis* sp. 32 in [Slos *et al.* (2017)](https://paperpile.com/c/f6CXok/OzZ1t)

The type isolate by present designation is SB454, deposited at the *Caenorhabditis* Genetics Center. The species reproduces through females and males. The species is delineated and diagnosed by the fertile cross with the type isolate SB454 in both cross directions, yielding highly fertile hybrid females and males that are interfertile and cross-fertile with their parent strains. The species reproduces through males and females. This species differs by SSU, LSU and ITS2 DNA sequences from all species listed in Tables 1 and 2 of Félix *et al.* (2014), *C. sinica* [(Huang *et al.* 2014)](https://paperpile.com/c/f6CXok/hGWP7), *C. monodelphis* [(Slos *et al.* 2017)](https://paperpile.com/c/f6CXok/OzZ1t), *C. dolen*s, *C. astrocarya* [(Ferrari *et al.* 2017)](https://paperpile.com/c/f6CXok/zqaoI) and Table 1 of the present article. Note that these ribosomal DNA sequences may vary within the species. From molecular data, the closest species is *C. afra,* with which it does not form any adult progeny (Table S2). The type isolate was isolated in February 2013 from the faeces of a millipede *Archispirostreptus gigas* (Peters, 1855) (Spirostreptidae) from East Africa bought in spring 2013 on an insect market in Berlin. See Fig. 4H, Fig. S6 and Fig. S7 for pictures of male genitalia. The fan is wide, closed anteriorly and the dorsal rays are in antero-posterior positions 5 and 7. The spicule tips are broad, as in *C. afra*, but broader than species of the Elegans group such as *C. zanzibari* (Fig. S8). The males mate in a parallel position. The species is named in honor of John Sulston (1942-2018) for his work on *C. elegans* cell lineage and genome sequence.

### *Caenorhabditis quiockensis* Stevens and Félix sp. n.

Zoobank identifier urn:lsid:zoobank.org:act:E96F15FF-3720-40E7-8684-7D5E47D72E43

= *Caenorhabditis* sp. 38 in [Slos *et al.* (2017)](https://paperpile.com/c/f6CXok/OzZ1t)

The type isolate by present designation is JU2745 deposited at the *Caenorhabditis* Genetics Center. The species reproduces through females and males. The species is delineated and diagnosed by the fertile cross with the type isolate JU2745 in both cross directions, yielding highly fertile hybrid females and males that are interfertile and cross-fertile with their parent strains. The species reproduces through males and females. This species differs by SSU, LSU and ITS2 DNA sequences from all species listed in Tables 1 and 2 of Félix *et al.* (2014), *C. sinica* [(Huang *et al.* 2014)](https://paperpile.com/c/f6CXok/hGWP7), *C. monodelphis* [(Slos *et al.* 2017)](https://paperpile.com/c/f6CXok/OzZ1t), *C. dolen*s, *C. astrocarya* [(Ferrari *et al.* 2017)](https://paperpile.com/c/f6CXok/zqaoI) and Table 1 of the present article. Note that these ribosomal DNA sequences may vary within the species. From molecular data, the closest species are *C. dolens, C. castelli* and *C. angaria,* with which it does not form any adult progeny (Table S2). The type isolate was isolated from a rotting fruit sampled in Guadeloupe (GPS 16.1761, -61.6851) on 9 June 2014. Other isolates were found on the neighbouring island of Dominica. See Fig. S6A-C for pictures of male genitalia. The fan is of reduced size (Fig. S6C) compared to *Elegans* supergroup species (the latter displayed in Fig. 4A-C and Fig. S7), with an oval shape, open anteriorly. The dorsal rays are in antero-posterior positions 4 and 7. The males mate in a spiral position. The species is named after its place of isolation, next to the Quiock river trail.

### *Caenorhabditis waitukubuli* Stevens and Félix sp. n.

Zoobank identifier

urn:lsid:zoobank.org:act:00DB134A-EF7C-4302-B531-6862BE3E1363

(= *Caenorhabditis* sp. 39)

The type isolate by present designation is NIC564, deposited at the *Caenorhabditis* Genetics Center. The species reproduces through females and males. The species is delineated and diagnosed by the fertile cross with the type isolate NIC564 in both cross directions, yielding highly fertile hybrid females and males that are interfertile and cross-fertile with their parent strains. The species reproduces through males and females. This species differs by SSU, LSU and ITS2 DNA sequences from all species listed in Tables 1 and 2 of Félix et al. 2014, *C. sinica* [*(Huang et al. 2014)*](https://paperpile.com/c/f6CXok/hGWP7), *C. monodelphis* [(Slos *et al.* 2017)](https://paperpile.com/c/f6CXok/OzZ1t), *C. dolen*s, *C. astrocarya* [(Ferrari *et al.* 2017)](https://paperpile.com/c/f6CXok/zqaoI) and Table 1 of the present article. Note that these ribosomal DNA sequences may vary within the species. From molecular data, the closest species are *C. yunquensis,* *C. macrosperma, C. nouraguensis, C. becei*, and *C. panamensis*, with which it does not form any adult progeny (Table S2). The type isolate was isolated from a rotting fruit sampled in Morne Trois Pitons, Dominica (GPS 15.37737, -61.33824) on 13 May 2014 (with other isolates found in other areas of the island). See Fig. S6 and Fig. S7 for pictures of male genitalia. The species is named after its place of isolation, Dominique, in the local Native American language.

### *Caenorhabditis tribulationis* Stevens and Félix sp. n.

Zoobank identifier

urn:lsid:zoobank.org:act:AD7F15A7-2D6A-49D1-A7D1-61438405D9F9

= *Caenorhabditis* sp. 40 in [(Slos *et al.* 2017)](https://paperpile.com/c/f6CXok/OzZ1t)

The type isolate by present designation is JU2774 deposited at the *Caenorhabditis* Genetics Center. The species reproduces through females and males. The species is delineated and diagnosed by the fertile cross with the type isolate JU2774 in both cross directions, yielding highly fertile hybrid females and males that are interfertile and cross-fertile with their parent strains. The species reproduces through males and females. This species differs by SSU, LSU and ITS2 DNA sequences from all species listed in Tables 1 and 2 of Félix *et al.* (2014), *C. sinica* [(Huang *et al.* 2014)](https://paperpile.com/c/f6CXok/hGWP7), *C. monodelphis* [(Slos *et al.* 2017)](https://paperpile.com/c/f6CXok/OzZ1t)*, C. dolen*s, *C. astrocarya* [(Ferrari *et al.* 2017)](https://paperpile.com/c/f6CXok/zqaoI) and Table 1 of the present article. Note that these ribosomal DNA sequences may vary within the species. From molecular data, the closest species are *C. sinica* and *C. zanzibari* with which it does not form any adult progeny (Table S2). The type isolate was isolated from humus sampled below the cathedral fig tree *Ficus destruens* by Danbulla Road, Queensland, Australia (GPS -17.1774, 145.6600) on 08 Aug 2014. Other isolates were found in Queensland. See Fig. 4B,G for pictures of the male genitalia. The fan is wide, closed anteriorly and the dorsal rays are in antero-posterior positions 5 and 7. The anterior margin of the hook shows a distinctive three-lobed shape that is shared with *C. sinica* [(Huang *et al.* 2014)](https://paperpile.com/c/f6CXok/hGWP7) and *C*. *zanzibari* (Fig. 4G). The males mate in a parallel position. The species is named after its place and context of isolation, next to Cape Tribulation in Australia.

### *Caenorhabditis vivipara* Stevens and Félix sp. n.

Zoobank identifier

urn:lsid:zoobank.org:act:D424A402-F0C2-43FC-918F-67D576D39CF2

= *Caenorhabditis* sp. 43

The type isolate by present designation is NIC1070 deposited at the *Caenorhabditis* Genetics Center. The species reproduces through females and males. The species is delineated and diagnosed by the fertile cross with the type isolate NIC1070 in both cross directions, yielding highly fertile hybrid females and males that are interfertile and cross-fertile with their parent strains. The species reproduces through males and females. This species differs by SSU, LSU and ITS2 DNA sequences from all species listed in Tables 1 and 2 of Félix et al. (2014), *C. sinica* [(Huang *et al.* 2014)](https://paperpile.com/c/f6CXok/hGWP7), *C. monodelphis* [(Slos *et al.* 2017)](https://paperpile.com/c/f6CXok/OzZ1t), *C. dolen*s, *C. astrocarya* [(Ferrari *et al.* 2017)](https://paperpile.com/c/f6CXok/zqaoI) and Table 1 of the present article. Note that these ribosomal DNA sequences may vary within the species. From molecular data, the closest species is *C. portoensis*, with which it does not form any adult progeny (Table S2). The type isolate was isolated from rotting red berries sampled in Chichén Itzá, Yucatán, Mexico (GPS 20.67859, -88.56941) on 17 Oct 2014. See Fig. S6E-I for pictures of a female adult and male genitalia. The male fan is wide and well-developed, with a serrated margin and no terminal notch. Dorsal rays are in antero-posterior positions 4 and 8. The males mate in a parallel position. The strain is viviparous: the embryos hatch within the mother and exit through the vulva as young L1 juveniles The species is named after this viviparity.
