# Supplementary material for: Comparative genomics of 10 new Caenorhabditis species
Source: Evol Lett. 2019 Apr 2;3(2):217–36. doi: 10.1002/evl3.110 (PMC6457397; doi:10.1002/evl3.110)
Supplement: Supplementary file 1 — Table S1. List of isolates and their origin. Table S2. Mating tests. This table contains several sheets, showing the results of crosses between isolates of different species. Successful crosses are labeled in green.“100s of embryos” refer to unhatched dead embryos remaining on the plate. Table S3. Detailed genome assembly and gene prediction statistics. Table S4. Morphological characters used for ancestral state reconstruction. ‘1’ denotes presence or existence; ‘0’ denotes absence. Table S5. Genome contents of C. sulstoni and C. elegans. Gene structure statistics were calculated using the longest isoform of each protein‐coding gene. UTR regions were not annotated in C. sulstoni and so were not considered in either species. Table S6. Genome statistics used in PGLS analysis. Gene structure statistics were calculated using the longest isoform of each protein‐coding gene. UTR regions were not considered as they were not annotated in several species. Repeat content was estimated de novo using RepeatModeler and RepeatMasker. Table S7. Mean branch lengths from Maximum likelihood gene tree of all Notch‐like proteins. Branch lengths were extracted using a custom Python script (available at https://github.com/lstevens17/caeno-ten-descriptions). Table S8. EGF‐like repeat counts for LIN‐12/GLP‐1 homologues. Counts of EGF‐like repeats were obtained from were obtained from the ProSiteProfiles database (release 2017_09). Table S9. Accessions and links to data used in phylogenomic analysis. Table S10. Completeness and duplication statistics for 28 Caenorhabditis species based on 8,286 orthologues. We selected groups of orthologues which were present in at least 22 species and had a mean count of 1. The duplication ratio was calculated by dividing the total number of sequences present for each species by the total number of orthogroups which contained a representative sequence for that species. Figure S1. Assembly spans and genome size estimates. Kmers of length 19 were counted usi [file EVL3-3-217-s001.zip › evl3110-sup-0001-SuppMat/evl3110-sup-0002-SuppMat.docx]

Detailed bioinformatics methods and versions

###

###

# 1. Genome assembly and gene prediction

### *Caenorhabditis parvicauda* (sp. 21)

We used FastQC to assess data quality and used Skewer to remove low quality bases and adapter sequences. Read originating from non-target organisms were identified and removed using taxon-annotated, GC-coverage plots generated by blobtools. Briefly, we generated a preliminary assembly using CLC Bio, and used Bowtie to map reads to the this assembly. Taxonomic annotations were obtained by searching the nt using NCBI BLAST+ (megablast) and UniProt References proteomes database using diamond (blastp). We counted kmers using KMC and assessed levels of heterozygosity and data quality using the resulting kmer spectra. We corrected sequencing errors using Bless. Filtered reads were assembled using Platanus. We assembled trimmed RNA-seq data using Trinity, and aligned the assembled transcripts to the Platanus assembly using BLAST+ (megablast), which was then scaffolded by SCUBAT2. A repeat library was constructed using known Rhabditida repeats obtained from RepBase and repeats that were identified *de novo* in the assembly using RepeatModeler, which were masked using RepeatMasker (interspersed repeats hard-masked; low-complexity repeats softmasked). Trimmed RNA-seq reads were aligned to the genome using STAR, and the resulting BAM file was provided to BRAKER for gene prediction. Genome assembly and gene prediction quality were assessed using CEGMA and BUSCO.

| **Software** | **Version** | **Relevant parameters** |
| --- | --- | --- |
| FastQC | v0.11.5 |  |
| KMC | v2.3 | -k19 |
| Bless | v1p01 | -kmerlength 21 |
| Skewer | 0.2.2 | -q 30 -l 61 |
| CLC assembler | 5.0.0 |  |
| CLC mapper | 4.2.1 | -l 0.9 -s 0.9 |
| BLASTn (NCBI-BLAST+) | 2.3 | -culling_limit 5 -evalue 1e-25 |
| blobtools | v0.9.19.1 |  |
| Bowtie | 2.2.2.4 |  |
| Platanus | 1.2.4 |  |
| SCUBAT | v2 | -max 20000 |
| RepeatModeler | open-1.0.8 |  |
| RepeatModeler | open-4.0.6 |  |
| STAR | 2.5.1b | --twopassMode Basic |
| BRAKER | v1.9 |  |
| BUSCO | 3.0.2 | -l nematoda_odb9 -m [genome\|proteins] |

**Table 1: Versions and parameters of software used to assemble the *C. parvicauda* genome.** Software was run using default parameters unless stated.

### *Caenorhabditis zanzibari* (sp. 26)

We used FastQC to assess data quality and used Skewer to remove low quality bases and adapter sequences. Read originating from non-target organisms were identified and removed using taxon-annotated, GC-coverage plots generated by blobtools. Briefly, we generated a preliminary assembly using CLC Bio, and used Bowtie to map reads to the this assembly. Taxonomic annotations were obtained by searching the nt using NCBI BLAST+ (megablast) and UniProt References proteomes database using diamond (blastp). We counted kmers using KMC and assessed levels of heterozygosity and data quality using the resulting kmer spectra. We corrected sequencing errors using Bless. Filtered reads were assembled using Platanus. We assembled trimmed RNA-seq data using Trinity, and aligned the assembled transcripts to the Platanus assembly using BLAST+ (megablast), which was then scaffolded by SCUBAT2. A repeat library was constructed using known Rhabditida repeats obtained from RepBase and repeats that were identified *de novo* in the assembly using RepeatModeler, which were masked using RepeatMasker (interspersed repeats hard-masked; low-complexity repeats softmasked). Trimmed RNA-seq reads were aligned to the genome using STAR, and the resulting BAM file was provided to BRAKER for gene prediction. Genome assembly and gene prediction quality were assessed using CEGMA and BUSCO.

| **Software** | **Version** | **Relevant parameters** |
| --- | --- | --- |
| FastQC | v0.11.5 |  |
| KMC | v2.3 | -k19 |
| Bless | v1p01 | -kmerlength 21 |
| Skewer | 0.2.2 | -q 30 -l 61 |
| CLC assembler | 5.0.0 |  |
| CLC mapper | 4.2.1 | -l 0.9 -s 0.9 |
| BLASTn (NCBI-BLAST+) | 2.3 | -culling_limit 5 -evalue 1e-25 |
| blobtools | v0.9.19.1 |  |
| Bowtie | 2.2.2.4 |  |
| Platanus | 1.2.4 |  |
| SCUBAT | v2 | -max 20000 |
| RepeatModeler | open-1.0.8 |  |
| RepeatModeler | open-4.0.6 |  |
| STAR | 2.5.1b | --twopassMode Basic |
| BRAKER | v1.9 |  |
| BUSCO | 3.0.2 | -l nematoda_odb9 -m [genome\|proteins] |

**Table 2: Versions and parameters of software used to assemble the *C. zanzibari* (sp. 26) genome.** Software was run using default parameters unless stated.

### *Caenorhabditis panamensis* (sp. 28)

We used FastQC to assess data quality and used FASTX Toolkit to remove low quality bases and adapter sequences. Adapters were removed from long-insert (or “mate-pair”) data using NextClip. Read originating from non-target organisms were identified and removed using Kraken. We corrected sequencing errors using Blue. Filtered reads (including long insert data) were assembled using Platanus. A repeat library was constructed using known Rhabditida repeats obtained from RepBase and repeats that were identified *de novo* in the assembly using RepeatModeler, which were masked using RepeatMasker (interspersed repeats were hard-masked; low-complexity repeats were softmasked). A preliminary gene set was predicted using MAKER (by supplying *C. elegans* N2 proteome and predictions from CEGMA as input), using evidence from SNAP and GeneMark-ES. The resulting gene set was used to train AUGUSTUS to generate final gene predictions. Genome assembly and gene prediction quality were assessed using CEGMA and BUSCO.

| **Software** | **Version** | **Relevant parameters** |
| --- | --- | --- |
| FastQC | 0.11.5 |  |
| FASTX-Toolkit fastx_clipper | 0.0.13 | -n -l 25 -M 9 -Q 33 |
| NextClip | 1.3 | -stage 2 (alignment against preliminary ABySS unitigs) |
| Kraken | 1.0 | NCBI nt built 10/06/2014 |
| Blue | 1.1.2 | Tessel: -k 31 -m 3  GenerateMerPairs: -m 3  Blue: -m 10 -paired -variable -g 90 |
| Platanus | 1.2.4 | Assemble: -c 29 -s 3 -u 0.05  Scaffolding: -s 35 -n 150 (short-insert library) -n 2000 (mate-pair library) |
| RepeatModeler | open-1.0.8 |  |
| RepeatModeler | open-4.0.6 |  |
| MAKER | 2.31 |  |
| SNAP | - |  |
| GeneMark-ES | 4.33 | --ES --max_intron 20000 |
| AUGUSTUS | 3.2.3 | --optrounds=3 |
| BUSCO | 3.0.2 | -l nematoda_odb9 -m [genome\|proteins] |

**Table 3: Versions and parameters of software used to assemble the *C. panamensis* (sp. 28) genome.** Software was run using default parameters unless stated.

### *Caenorhabditis becei* (sp. 29)

We used FastQC to assess data quality and used FASTX Toolkit to remove low quality bases and adapter sequences. Adapters were removed from long-insert (or “mate-pair”) data using NextClip. Read originating from non-target organisms were identified and removed using Kraken. We corrected sequencing errors using Blue. Filtered reads (including long insert data) were assembled using Platanus. A repeat library was constructed using known Rhabditida repeats obtained from RepBase and repeats that were identified *de novo* in the assembly using RepeatModeler, which were masked using RepeatMasker (interspersed repeats were hard-masked; low-complexity repeats were softmasked). A preliminary gene set was predicted using MAKER (by supplying *C. elegans* N2 proteome and predictions from CEGMA as input), using evidence from SNAP and GeneMark-ES. The resulting gene set was used to train AUGUSTUS to generate final gene predictions. Genome assembly and gene prediction quality were assessed using CEGMA and BUSCO.

| **Software** | **Version** | **Relevant parameters** |
| --- | --- | --- |
| FastQC | 0.11.5 |  |
| Trimmer X | 0.0.13 | -n -l 25 -M 9 -Q 33 |
| NextClip | 1.3 | -stage 2 (alignment against preliminary ABySS unitigs) |
| Kraken | 1.0 | NCBI nt built 10/06/2014 |
| Blue | 1.1.2 | Tessel: -k 31 -m 3  GenerateMerPairs: -m 3  Blue: -m 10 -paired -variable -g 90 |
| Platanus | 1.2.4 | Assemble: -c 29 -s 3 -u 0.05  Scaffolding: -s 35 -n 150 (short-insert library) -n 2000 (mate-pair library) |
| RepeatModeler | open-1.0.8 |  |
| RepeatModeler | open-4.0.6 |  |
| MAKER | 2.31 |  |
| CEGMA | 2.9 |  |
| SNAP | - |  |
| GeneMark-ES | 4.33 | --ES --max_intron 20000 |
| AUGUSTUS | 3.2.3 | --optrounds=3 |
| BUSCO | 3.0.2 | -l nematoda_odb9 -m [genome\|proteins] |

**Table 4: Versions and parameters of software used to assemble the *C. becei* (sp. 29) genome.** Software was run using default parameters unless stated.

### *Caenorhabditis uteleia* (sp. 31)

We used FastQC to assess data quality and used Skewer to remove low quality bases and adapter sequences. Read originating from non-target organisms were identified and removed using taxon-annotated, GC-coverage plots generated by blobtools. Briefly, we generated a preliminary assembly using CLC Bio, and used Bowtie to map reads to the this assembly. Taxonomic annotations were obtained by searching the nt using NCBI BLAST+ (megablast) and UniProt References proteomes database using diamond (blastp). We estimated the optimal kmer length for assembly using Kmer-genie. We counted kmers using KMC and assessed levels of heterozygosity and data quality using the resulting kmer spectra. We corrected sequencing errors using Bless. Filtered reads were assembled using Velvet. We assembled trimmed RNA-seq data using Trinity, and aligned the assembled transcripts to the Velvet assembly using BLAST+ (megablast), which was then scaffolded by SCUBAT2. A repeat library was constructed using known Rhabditida repeats obtained from RepBase and repeats that were identified *de novo* in the assembly using RepeatModeler, which were masked using RepeatMasker (interspersed repeats hard-masked; low-complexity repeats softmasked). Trimmed RNA-seq reads were aligned to the genome using STAR, and the resulting BAM file was provided to BRAKER for gene prediction. Genome assembly and gene prediction quality were assessed using CEGMA and BUSCO.

| **Software** | **Version** | **Relevant parameters** |
| --- | --- | --- |
| FastQC | v0.11.5 |  |
| KMC | v2.3 | -k19 |
| Bless | v1p01 | -kmerlength 21 |
| Skewer | 0.2.2 | -q 30 -l 61 |
| CLC assembler | 5.0.0 |  |
| CLC mapper | 4.2.1 | -l 0.9 -s 0.9 |
| BLASTn (NCBI-BLAST+) | 2.3 | -culling_limit 5 -evalue 1e-25 |
| blobtools | v0.9.19.1 |  |
| Bowtie | 2.2.2.4 |  |
| Kmer-genie | 1.6982 |  |
| Velvet | 1.2.10 | 107 -exp_cov auto -cov_cutoff auto |
| Trinity | 2.2.0 |  |
| SCUBAT | v2 | -max 20000 |
| RepeatModeler | open-1.0.8 |  |
| RepeatModeler | open-4.0.6 |  |
| STAR | 2.5.1b | --twopassMode Basic |
| BRAKER | v1.9 |  |
| BUSCO | 3.0.2 | -l nematoda_odb9 -m [genome\|proteins] |

**Table 5: Versions and parameters of software used to assemble the *C. uteleia* (sp. 31) genome.** Software was run using default parameters unless stated.

### *Caenorhabditis sulstoni* (sp. 32)

We used FastQC to assess data quality and used Skewer to remove low quality bases and adapter sequences. Read originating from non-target organisms were identified and removed using taxon-annotated, GC-coverage plots generated by blobtools. Briefly, we generated a preliminary assembly using CLC Bio, and used Bowtie to map reads to the this assembly. Taxonomic annotations were obtained by searching the nt using NCBI BLAST+ (megablast) and UniProt References proteomes database using diamond (blastp). We counted kmers using KMC and assessed levels of heterozygosity and data quality using the resulting kmer spectra. We corrected sequencing errors using Bless. Filtered reads were assembled using Velvet. We assembled trimmed RNA-seq data using Trinity, and aligned the assembled transcripts to the Velvet assembly using BLAST+ (megablast), which was then scaffolded by SCUBAT2. A repeat library was constructed using known Rhabditida repeats obtained from RepBase and repeats that were identified *de novo* in the assembly using RepeatModeler, which were masked using RepeatMasker (interspersed repeats hard-masked; low-complexity repeats softmasked). Trimmed RNA-seq reads were aligned to the genome using STAR, and the resulting BAM file was provided to BRAKER for gene prediction. Genome assembly and gene prediction quality were assessed using CEGMA and BUSCO.

| **Software** | **Version** | **Relevant parameters** |
| --- | --- | --- |
| FastQC | v0.11.5 |  |
| KMC | v2.3 | -k19 |
| Bless | v1p01 | -kmerlength 21 |
| Skewer | 0.2.2 | -q 30 -l 61 |
| CLC assembler | 5.0.0 |  |
| CLC mapper | 4.2.1 | -l 0.9 -s 0.9 |
| BLASTn (NCBI-BLAST+) | 2.3 | -culling_limit 5 -evalue 1e-25 |
| blobtools | v0.9.19.1 |  |
| Bowtie | 2.2.2.4 |  |
| Velvet | 1.2.10 | 107 -exp_cov auto -cov_cutoff auto |
| Trinity | 2.2.0 |  |
| SCUBAT | v2 | -max 20000 |
| RepeatModeler | open-1.0.8 |  |
| RepeatModeler | open-4.0.6 |  |
| STAR | 2.5.1b | --twopassMode Basic |
| BRAKER | v1.9 |  |
| BUSCO | 3.0.2 | -l nematoda_odb9 -m [genome\|proteins] |

**Table 6: Versions and parameters of software used to assemble the *C. sulstoni* (sp. 32) genome.** Software was run using default parameters unless stated.

### *Caenorhabditis quiockensis* (sp. 38)

We used FastQC to assess data quality and used Skewer to remove low quality bases and adapter sequences. Read originating from non-target organisms were identified and removed using taxon-annotated, GC-coverage plots generated by blobtools. Briefly, we generated a preliminary assembly using CLC Bio, and used Bowtie to map reads to the this assembly. Taxonomic annotations were obtained by searching the nt using NCBI BLAST+ (megablast) and UniProt References proteomes database using diamond (blastp). We counted kmers using KMC and assessed levels of heterozygosity and data quality using the resulting kmer spectra. We corrected sequencing errors using Bless. Filtered reads were assembled using Velvet. We assembled trimmed RNA-seq data using Trinity, and aligned the assembled transcripts to the Velvet assembly using BLAST+ (megablast), which was then scaffolded by SCUBAT2. A repeat library was constructed using known Rhabditida repeats obtained from RepBase and repeats that were identified *de novo* in the assembly using RepeatModeler, which were masked using RepeatMasker (interspersed repeats hard-masked; low-complexity repeats softmasked). Trimmed RNA-seq reads were aligned to the genome using STAR, and the resulting BAM file was provided to BRAKER for gene prediction. Genome assembly and gene prediction quality were assessed using CEGMA and BUSCO.

| **Software** | **Version** | **Relevant parameters** |
| --- | --- | --- |
| FastQC | v0.11.5 |  |
| KMC | v2.3 | -k19 |
| Bless | v1p01 | -kmerlength 21 |
| Skewer | 0.2.2 | -q 30 -l 61 |
| CLC assembler | 5.0.0 |  |
| CLC mapper | 4.2.1 | -l 0.9 -s 0.9 |
| BLASTn (NCBI-BLAST+) | 2.3 | -culling_limit 5 -evalue 1e-25 |
| blobtools | v0.9.19.1 |  |
| Bowtie | 2.2.2.4 |  |
| Velvet | 1.2.10 |  |
| Trinity | 2.2.0 |  |
| SCUBAT | v2 | -max 20000 |
| RepeatModeler | open-1.0.8 |  |
| RepeatModeler | open-4.0.6 |  |
| STAR | 2.5.1b | --twopassMode Basic |
| BRAKER | v1.9 |  |
| BUSCO | 3.0.2 | -l nematoda_odb9 -m [genome\|proteins] |

**Table 7: Versions and parameters of software used to assemble the *C. quiockensis* (sp. 38) genome.** Software was run using default parameters unless stated.

### *Caenorhabditis waitukubuli* (sp. 39)

We used FastQC to assess data quality and used Skewer to remove low quality bases and adapter sequences. Read originating from non-target organisms were identified and removed using taxon-annotated, GC-coverage plots generated by blobtools. Briefly, we generated a preliminary assembly using CLC Bio, and used Bowtie to map reads to the this assembly. Taxonomic annotations were obtained by searching the nt using NCBI BLAST+ (megablast) and UniProt References proteomes database using diamond (blastp). We counted kmers using KMC and assessed levels of heterozygosity and data quality using the resulting kmer spectra. We corrected sequencing errors using Bless. Filtered reads were assembled using Platanus. We assembled trimmed RNA-seq data using Trinity, and aligned the assembled transcripts to the Platanus assembly using BLAST+ (megablast), which was then scaffolded by SCUBAT2. A repeat library was constructed using known Rhabditida repeats obtained from RepBase and repeats that were identified *de novo* in the assembly using RepeatModeler, which were masked using RepeatMasker (interspersed repeats hard-masked; low-complexity repeats softmasked). Trimmed RNA-seq reads were aligned to the genome using STAR, and the resulting BAM file was provided to BRAKER for gene prediction. Genome assembly and gene prediction quality were assessed using CEGMA and BUSCO.

| **Software** | **Version** | **Relevant parameters** |
| --- | --- | --- |
| FastQC | v0.11.5 |  |
| KMC | v2.3 | -k19 |
| Bless | v1p01 | -kmerlength 21 |
| Skewer | 0.2.2 | -q 30 -l 61 |
| CLC assembler | 5.0.0 |  |
| CLC mapper | 4.2.1 | -l 0.9 -s 0.9 |
| BLASTn (NCBI-BLAST+) | 2.3 | -culling_limit 5 -evalue 1e-25 |
| blobtools | v0.9.19.1 |  |
| Bowtie | 2.2.2.4 |  |
| Platanus | 1.2.4 |  |
| Trinity | 2.2.0 |  |
| SCUBAT | v2 | -max 20000 |
| RepeatModeler | open-1.0.8 |  |
| RepeatModeler | open-4.0.6 |  |
| STAR | 2.5.1b | --twopassMode Basic |
| BRAKER | v1.9 |  |
| BUSCO | 3.0.2 | -l nematoda_odb9 -m [genome\|proteins] |

**Table 8: Versions and parameters of software used to assemble the *C. waitukubuli* (sp. 39) genome.** Software was run using default parameters unless stated.

### *Caenorhabditis tribulationis* (sp. 40)

We used FastQC to assess data quality and used Skewer to remove low quality bases and adapter sequences. Read originating from non-target organisms were identified and removed using taxon-annotated, GC-coverage plots generated by blobtools. Briefly, we generated a preliminary assembly using CLC Bio, and used Bowtie to map reads to the this assembly. Taxonomic annotations were obtained by searching the nt using NCBI BLAST+ (megablast) and UniProt References proteomes database using diamond (blastp). We counted kmers using KMC and assessed levels of heterozygosity and data quality using the resulting kmer spectra. We corrected sequencing errors using Bless. Filtered reads were assembled using Velvet. We assembled trimmed RNA-seq data using Trinity, and aligned the assembled transcripts to the Velvet assembly using BLAST+ (megablast), which was then scaffolded by SCUBAT2. A repeat library was constructed using known Rhabditida repeats obtained from RepBase and repeats that were identified *de novo* in the assembly using RepeatModeler, which were masked using RepeatMasker (interspersed repeats hard-masked; low-complexity repeats softmasked). Trimmed RNA-seq reads were aligned to the genome using STAR, and the resulting BAM file was provided to BRAKER for gene prediction. Genome assembly and gene prediction quality were assessed using CEGMA and BUSCO.

| **Software** | **Version** | **Relevant parameters** |
| --- | --- | --- |
| FastQC | v0.11.5 |  |
| KMC | v2.3 | -k19 |
| Bless | v1p01 | -kmerlength 21 |
| Skewer | 0.2.2 | -q 30 -l 61 |
| CLC assembler | 5.0.0 |  |
| CLC mapper | 4.2.1 | -l 0.9 -s 0.9 |
| BLASTn (NCBI-BLAST+) | 2.3 | -culling_limit 5 -evalue 1e-25 |
| blobtools | v0.9.19.1 |  |
| Velvet | 1.2.10 |  |
| Platanus | 1.2.4 |  |
| Trinity | 2.2.0 |  |
| SCUBAT | v2 | -max 20000 |
| RepeatModeler | open-1.0.8 |  |
| RepeatModeler | open-4.0.6 |  |
| STAR | 2.5.1b | --twopassMode Basic |
| BRAKER | v1.9 |  |
| BUSCO | 3.0.2 | -l nematoda_odb9 -m [genome\|proteins] |

**Table 9: Versions and parameters of software used to assemble the *C. tribulationis* (sp. 40) genome.** Software was run using default parameters unless stated.

### *Caenorhabditis vivipara* (sp. 43)

We used FastQC to assess data quality and used Skewer to remove low quality bases and adapter sequences. We counted kmers using KMC and assessed levels of heterozygosity and data quality using the resulting kmer spectra. Read originating from non-target organisms were identified and removed using taxon-annotated, GC-coverage plots generated by blobtools. Briefly, we generated a preliminary assembly using CLC Bio, and used Bowtie to map reads to the this assembly. Taxonomic annotations were obtained by searching the nt using NCBI BLAST+ (megablast) and UniProt References proteomes database using diamond (blastp). We counted kmers using KMC and assessed levels of heterozygosity and data quality using the resulting kmer spectra. Due to a lack of a distinct peak in the kmer spectra and low contiguity of the preliminary assembly, we decided not proceed with genome assembly. To obtain protein sequences for phylogenomic analyses, we assembled trimmed RNA-seq data using Trinity and predicted open reading frames in the assembled transcripts using TransDecoder.

| **Software** | **Version** | **Relevant parameters** |
| --- | --- | --- |
| FastQC | v0.11.5 |  |
| KMC | v2.3 | -k19 |
| Bless | v1p01 | -kmerlength 21 |
| Skewer | 0.2.2 | -q 30 -l 61 |
| CLC assembler | 5.0.0 |  |
| CLC mapper | 4.2.1 | -l 0.9 -s 0.9 |
| BLASTn (NCBI-BLAST+) | 2.3 | -culling_limit 5 -evalue 1e-25 |
| blobtools | v0.9.19.1 |  |
| Bowtie | 2.2.2.4 |  |
| Trinity | 2.2.0 |  |
| TransDecoder | v3.0.1 |  |

**Table 10: Versions and parameters of software used to assemble the *C. vivipara* (sp. 43) genome.** Software was run using default parameters unless stated.

# 2. Phylogenomic analyses

| **Software** | **Version** | **Relevant parameters** |
| --- | --- | --- |
| OrthoFinder | v1.1.4 | -og |
| FSA | 1.15.9 |  |
| RAxML | 8.2.10 | For gene trees:  -m PROTGAMMAAUTO -f a -# 100  For species tree:  -m PROTGAMMAGTR -f a -# 100 |
| PhyloTreePruner | v20150918 | 0.5 (bootstrap collapse threshold) |
| ASTRAL-III | v5.5.4 |  |
| trimAL | v1.4.rev15 | -gt 0.8 -st 0.001 -resoverlap 0.75 -seqoverlap 80 |
| catfasta2phyml.pl |  | -f -c |
| PhyloBayes MPI | 1.8 |  |
| Tracer | 1.6 |  |

**Table 11: Versions and parameters of software used in phylogenomic analyses.** Software was run using default parameters unless stated.

# 3. Genome size analysis

| **Software/Package** | **Version** | **Relevant parameters** |
| --- | --- | --- |
| RepeatModeler | open-1.0.8 |  |
| RepeatMasker | open-4.0.6 |  |
| ape | 5.2 | gls([feature] ~ genome_size, correlation = corBrownian(phy = caenoTree), data = caenoData, method = "ML") |

**Table 12: Versions and parameters of software used in genome size analysis.** Software was run using default parameters unless stated.

# 4. Notch-receptor analysis

| **Software** | **Version** | **Relevant parameters** |
| --- | --- | --- |
| OrthoFinder | v1.1.4 | -og |
| FSA | 1.15.9 |  |
| InterProScan | 5.27-66.0 |  |
| RAxML | 8.2.10 | -m PROTGAMMAAUTO -f a -# 100 |
| phytools | 0.6.60 | make.simmap(caenoTree,EGFcounts,model="ER",nsim=1000) |

**Table 13: Versions and parameters of software used in Notch-receptor analysis.** Software was run using default parameters unless stated.
