# Supplementary figures and images for: Comparative genomics of 10 new Caenorhabditis species
Source: Evol Lett. 2019 Apr 2;3(2):217–36. doi: 10.1002/evl3.110 (PMC6457397; doi:10.1002/evl3.110)

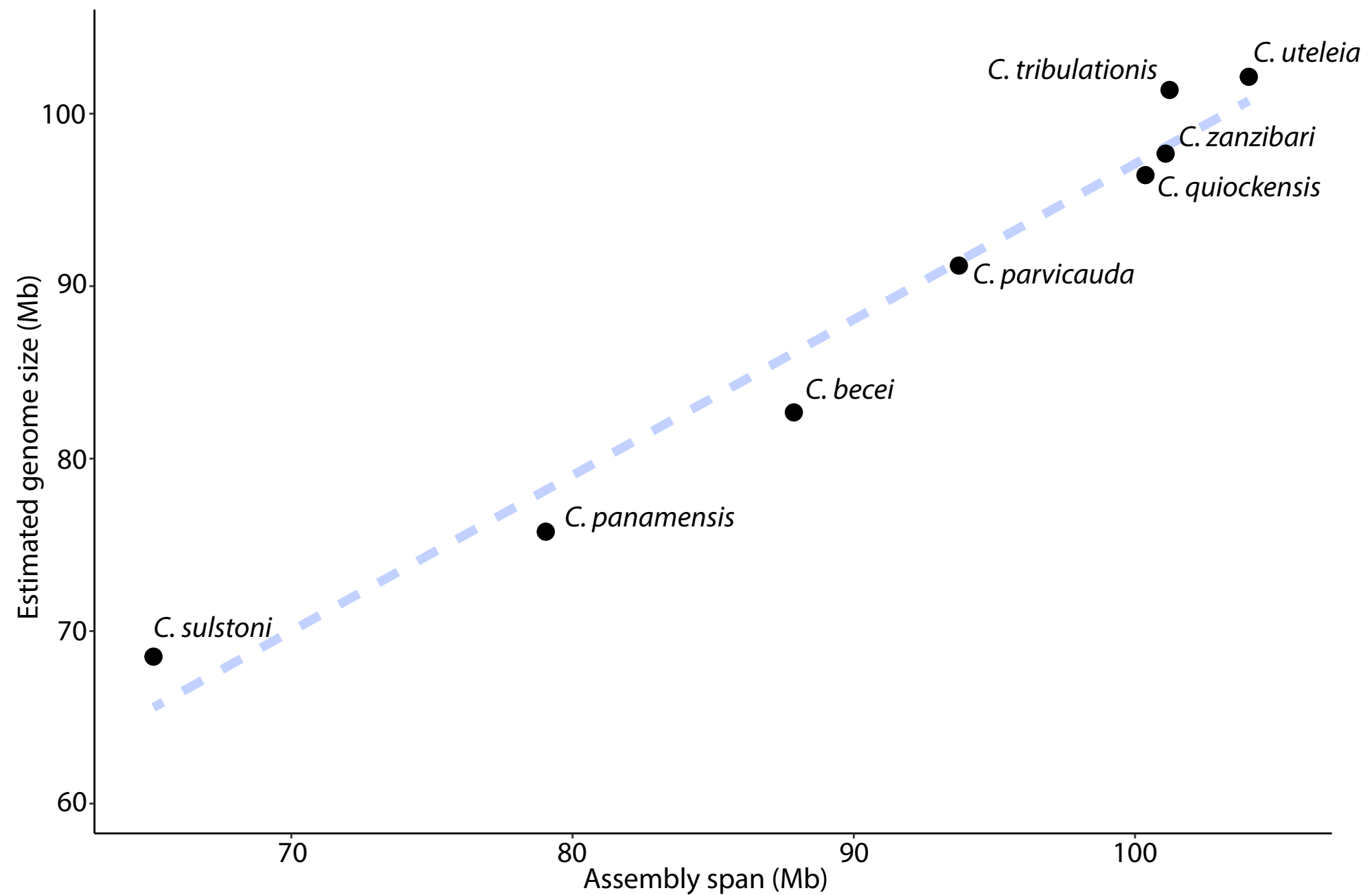

Supplement: Supplementary file 1 — Table S1. List of isolates and their origin. Table S2. Mating tests. This table contains several sheets, showing the results of crosses between isolates of different species. Successful crosses are labeled in green.“100s of embryos” refer to unhatched dead embryos remaining on the plate. Table S3. Detailed genome assembly and gene prediction statistics. Table S4. Morphological characters used for ancestral state reconstruction. ‘1’ denotes presence or existence; ‘0’ denotes absence. Table S5. Genome contents of C. sulstoni and C. elegans. Gene structure statistics were calculated using the longest isoform of each protein‐coding gene. UTR regions were not annotated in C. sulstoni and so were not considered in either species. Table S6. Genome statistics used in PGLS analysis. Gene structure statistics were calculated using the longest isoform of each protein‐coding gene. UTR regions were not considered as they were not annotated in several species. Repeat content was estimated de novo using RepeatModeler and RepeatMasker. Table S7. Mean branch lengths from Maximum likelihood gene tree of all Notch‐like proteins. Branch lengths were extracted using a custom Python script (available at https://github.com/lstevens17/caeno-ten-descriptions). Table S8. EGF‐like repeat counts for LIN‐12/GLP‐1 homologues. Counts of EGF‐like repeats were obtained from were obtained from the ProSiteProfiles database (release 2017_09). Table S9. Accessions and links to data used in phylogenomic analysis. Table S10. Completeness and duplication statistics for 28 Caenorhabditis species based on 8,286 orthologues. We selected groups of orthologues which were present in at least 22 species and had a mean count of 1. The duplication ratio was calculated by dividing the total number of sequences present for each species by the total number of orthogroups which contained a representative sequence for that species. Figure S1. Assembly spans and genome size estimates. Kmers of length 19 were counted usi [file EVL3-3-217-s001.zip › evl3110-sup-0001-SuppMat/evl3110-sup-0003-FigureS1.pdf]

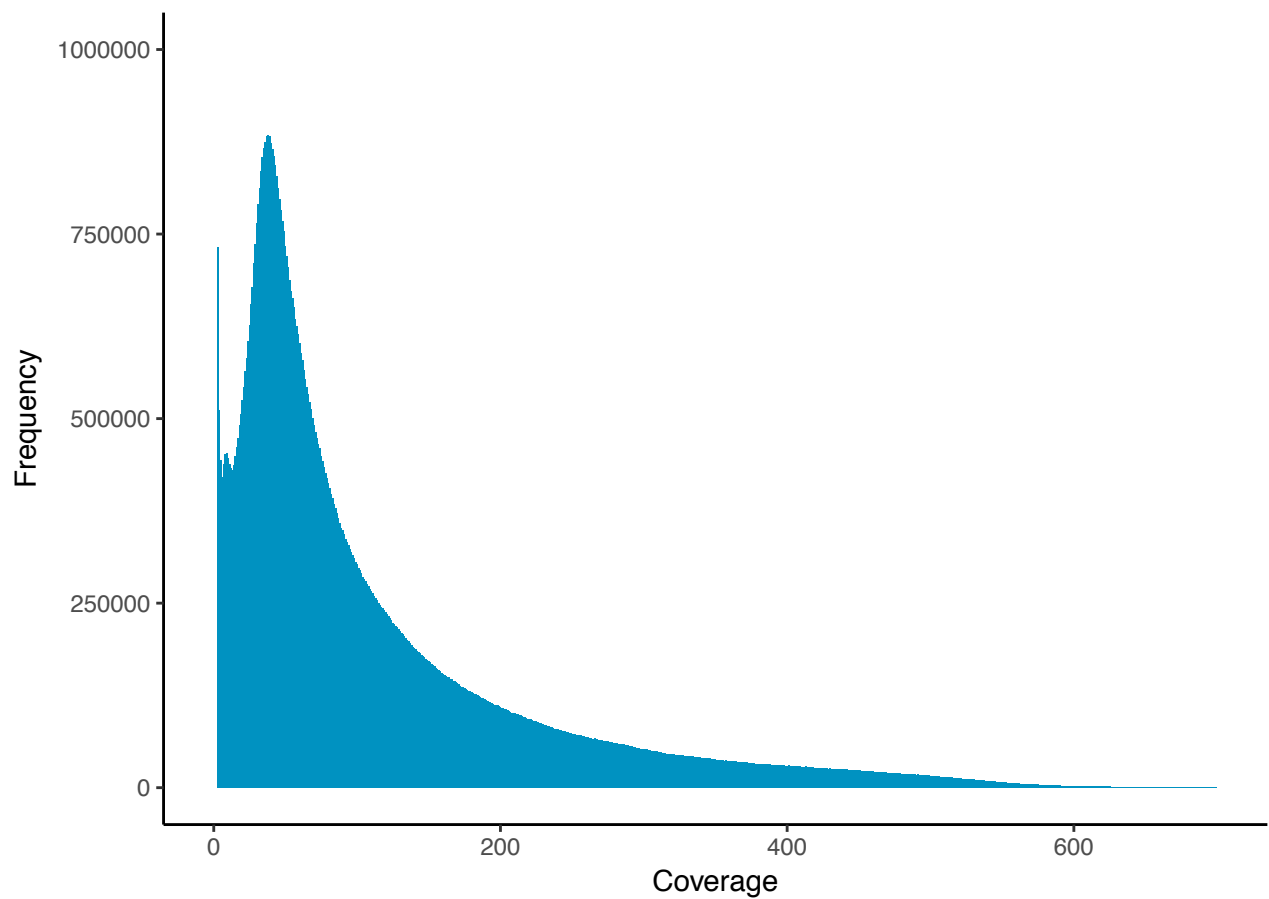

Supplement: Supplementary file 1 — Table S1. List of isolates and their origin. Table S2. Mating tests. This table contains several sheets, showing the results of crosses between isolates of different species. Successful crosses are labeled in green.“100s of embryos” refer to unhatched dead embryos remaining on the plate. Table S3. Detailed genome assembly and gene prediction statistics. Table S4. Morphological characters used for ancestral state reconstruction. ‘1’ denotes presence or existence; ‘0’ denotes absence. Table S5. Genome contents of C. sulstoni and C. elegans. Gene structure statistics were calculated using the longest isoform of each protein‐coding gene. UTR regions were not annotated in C. sulstoni and so were not considered in either species. Table S6. Genome statistics used in PGLS analysis. Gene structure statistics were calculated using the longest isoform of each protein‐coding gene. UTR regions were not considered as they were not annotated in several species. Repeat content was estimated de novo using RepeatModeler and RepeatMasker. Table S7. Mean branch lengths from Maximum likelihood gene tree of all Notch‐like proteins. Branch lengths were extracted using a custom Python script (available at https://github.com/lstevens17/caeno-ten-descriptions). Table S8. EGF‐like repeat counts for LIN‐12/GLP‐1 homologues. Counts of EGF‐like repeats were obtained from were obtained from the ProSiteProfiles database (release 2017_09). Table S9. Accessions and links to data used in phylogenomic analysis. Table S10. Completeness and duplication statistics for 28 Caenorhabditis species based on 8,286 orthologues. We selected groups of orthologues which were present in at least 22 species and had a mean count of 1. The duplication ratio was calculated by dividing the total number of sequences present for each species by the total number of orthogroups which contained a representative sequence for that species. Figure S1. Assembly spans and genome size estimates. Kmers of length 19 were counted usi [file EVL3-3-217-s001.zip › evl3110-sup-0001-SuppMat/evl3110-sup-0004-FigureS1.pdf]

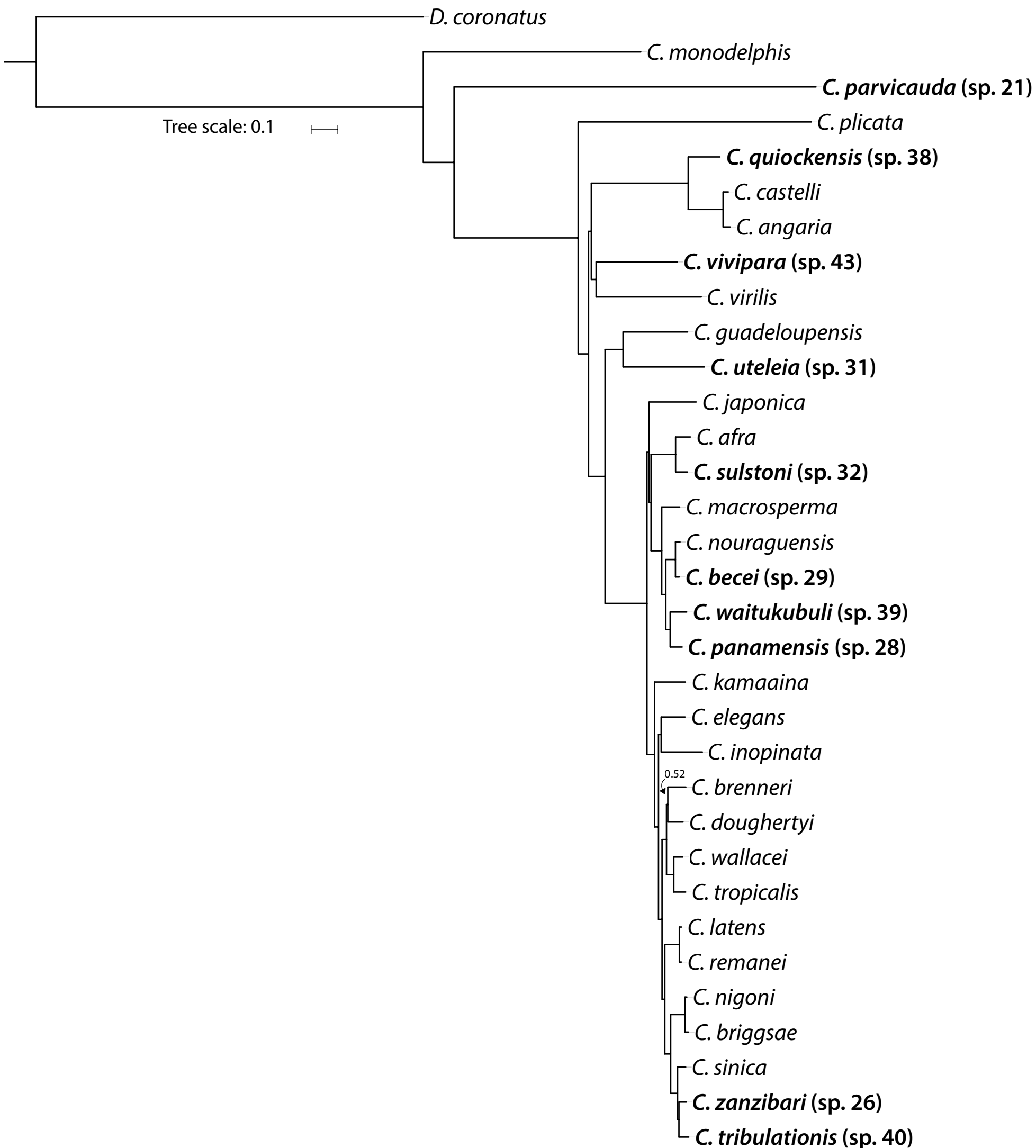

Supplement: Supplementary file 1 — Table S1. List of isolates and their origin. Table S2. Mating tests. This table contains several sheets, showing the results of crosses between isolates of different species. Successful crosses are labeled in green.“100s of embryos” refer to unhatched dead embryos remaining on the plate. Table S3. Detailed genome assembly and gene prediction statistics. Table S4. Morphological characters used for ancestral state reconstruction. ‘1’ denotes presence or existence; ‘0’ denotes absence. Table S5. Genome contents of C. sulstoni and C. elegans. Gene structure statistics were calculated using the longest isoform of each protein‐coding gene. UTR regions were not annotated in C. sulstoni and so were not considered in either species. Table S6. Genome statistics used in PGLS analysis. Gene structure statistics were calculated using the longest isoform of each protein‐coding gene. UTR regions were not considered as they were not annotated in several species. Repeat content was estimated de novo using RepeatModeler and RepeatMasker. Table S7. Mean branch lengths from Maximum likelihood gene tree of all Notch‐like proteins. Branch lengths were extracted using a custom Python script (available at https://github.com/lstevens17/caeno-ten-descriptions). Table S8. EGF‐like repeat counts for LIN‐12/GLP‐1 homologues. Counts of EGF‐like repeats were obtained from were obtained from the ProSiteProfiles database (release 2017_09). Table S9. Accessions and links to data used in phylogenomic analysis. Table S10. Completeness and duplication statistics for 28 Caenorhabditis species based on 8,286 orthologues. We selected groups of orthologues which were present in at least 22 species and had a mean count of 1. The duplication ratio was calculated by dividing the total number of sequences present for each species by the total number of orthogroups which contained a representative sequence for that species. Figure S1. Assembly spans and genome size estimates. Kmers of length 19 were counted usi [file EVL3-3-217-s001.zip › evl3110-sup-0001-SuppMat/evl3110-sup-0005-FigureS1.pdf]

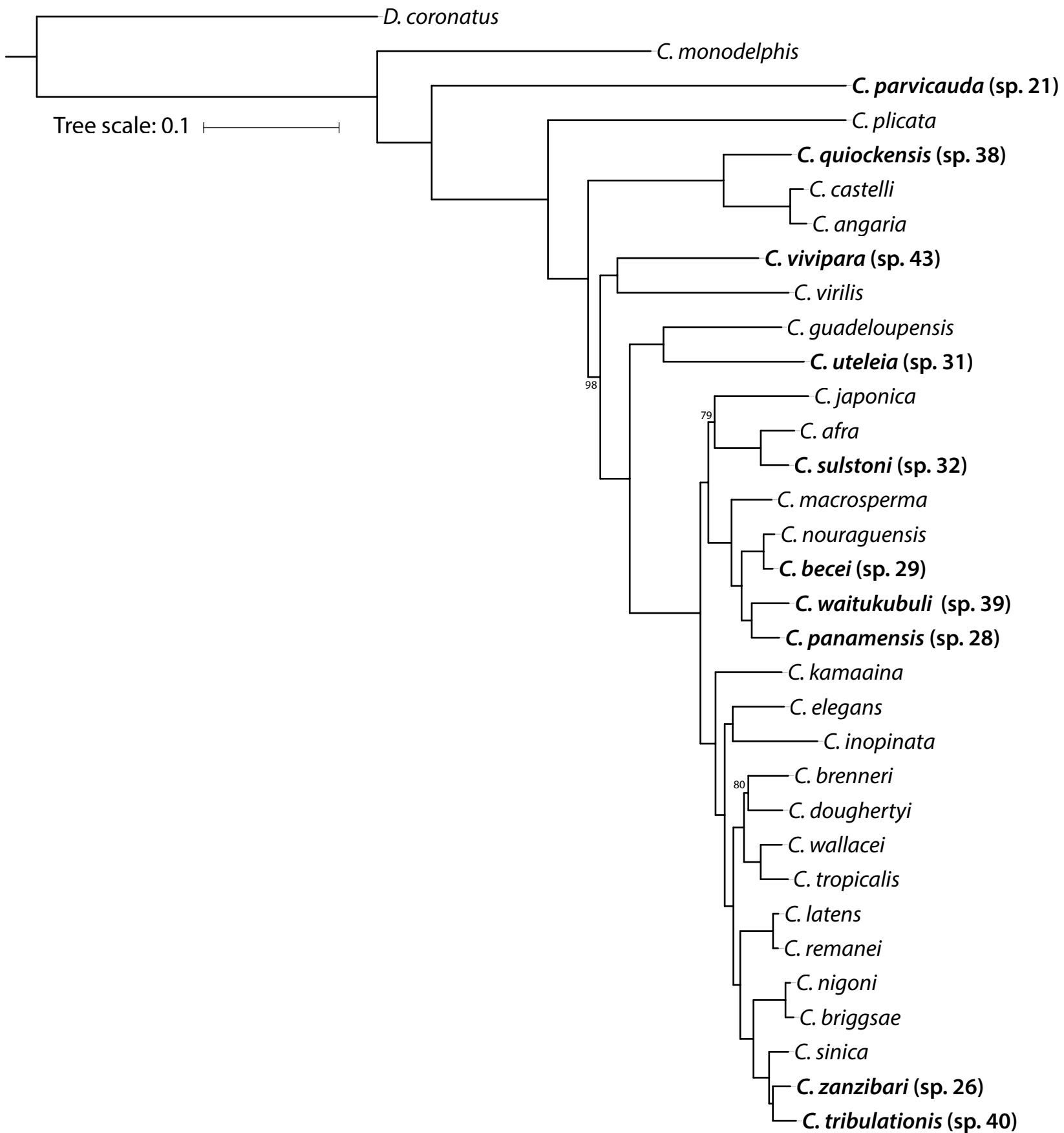

Supplement: Supplementary file 1 — Table S1. List of isolates and their origin. Table S2. Mating tests. This table contains several sheets, showing the results of crosses between isolates of different species. Successful crosses are labeled in green.“100s of embryos” refer to unhatched dead embryos remaining on the plate. Table S3. Detailed genome assembly and gene prediction statistics. Table S4. Morphological characters used for ancestral state reconstruction. ‘1’ denotes presence or existence; ‘0’ denotes absence. Table S5. Genome contents of C. sulstoni and C. elegans. Gene structure statistics were calculated using the longest isoform of each protein‐coding gene. UTR regions were not annotated in C. sulstoni and so were not considered in either species. Table S6. Genome statistics used in PGLS analysis. Gene structure statistics were calculated using the longest isoform of each protein‐coding gene. UTR regions were not considered as they were not annotated in several species. Repeat content was estimated de novo using RepeatModeler and RepeatMasker. Table S7. Mean branch lengths from Maximum likelihood gene tree of all Notch‐like proteins. Branch lengths were extracted using a custom Python script (available at https://github.com/lstevens17/caeno-ten-descriptions). Table S8. EGF‐like repeat counts for LIN‐12/GLP‐1 homologues. Counts of EGF‐like repeats were obtained from were obtained from the ProSiteProfiles database (release 2017_09). Table S9. Accessions and links to data used in phylogenomic analysis. Table S10. Completeness and duplication statistics for 28 Caenorhabditis species based on 8,286 orthologues. We selected groups of orthologues which were present in at least 22 species and had a mean count of 1. The duplication ratio was calculated by dividing the total number of sequences present for each species by the total number of orthogroups which contained a representative sequence for that species. Figure S1. Assembly spans and genome size estimates. Kmers of length 19 were counted usi [file EVL3-3-217-s001.zip › evl3110-sup-0001-SuppMat/evl3110-sup-0006-FigureS1.pdf]

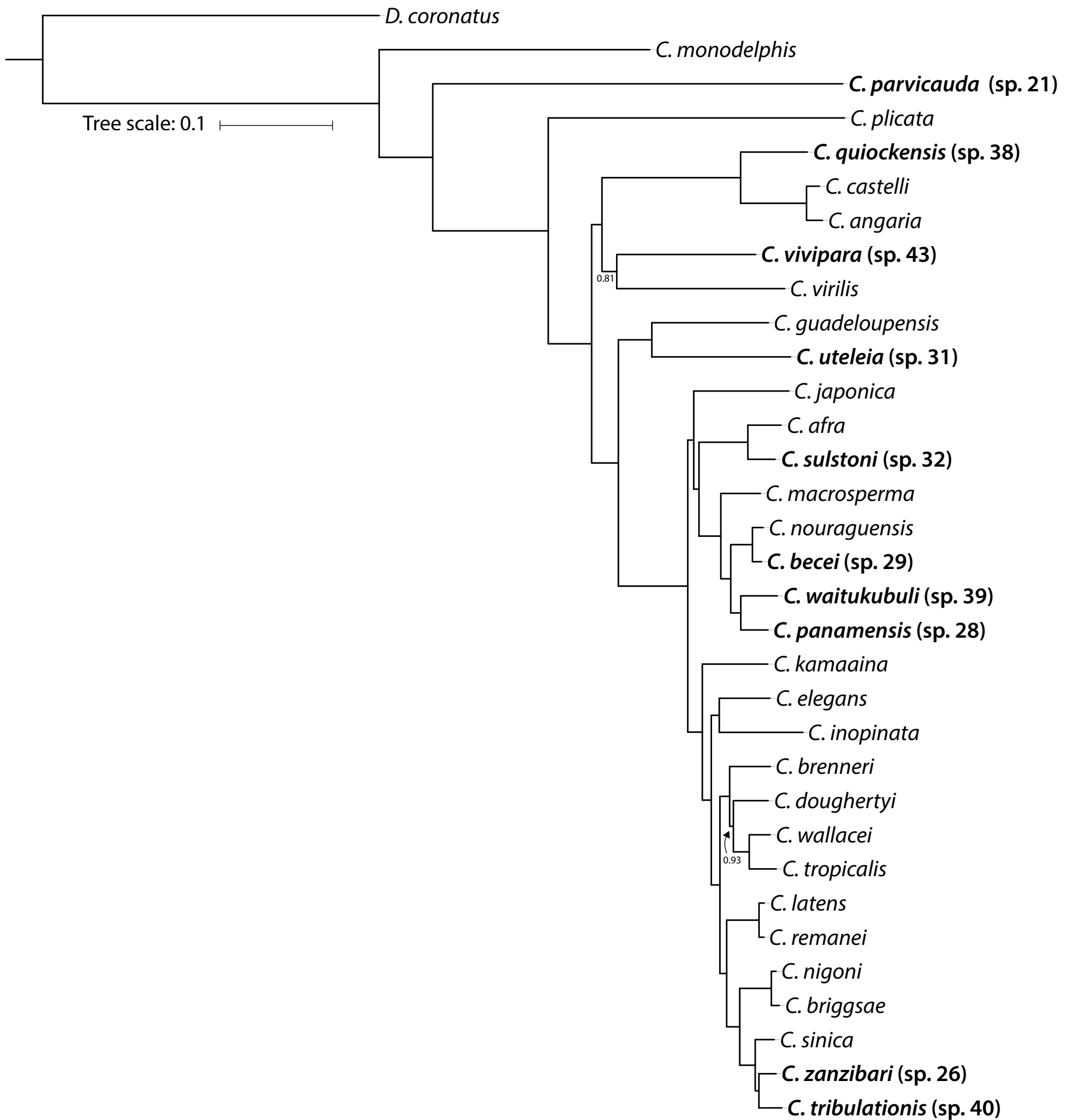

Supplement: Supplementary file 1 — Table S1. List of isolates and their origin. Table S2. Mating tests. This table contains several sheets, showing the results of crosses between isolates of different species. Successful crosses are labeled in green.“100s of embryos” refer to unhatched dead embryos remaining on the plate. Table S3. Detailed genome assembly and gene prediction statistics. Table S4. Morphological characters used for ancestral state reconstruction. ‘1’ denotes presence or existence; ‘0’ denotes absence. Table S5. Genome contents of C. sulstoni and C. elegans. Gene structure statistics were calculated using the longest isoform of each protein‐coding gene. UTR regions were not annotated in C. sulstoni and so were not considered in either species. Table S6. Genome statistics used in PGLS analysis. Gene structure statistics were calculated using the longest isoform of each protein‐coding gene. UTR regions were not considered as they were not annotated in several species. Repeat content was estimated de novo using RepeatModeler and RepeatMasker. Table S7. Mean branch lengths from Maximum likelihood gene tree of all Notch‐like proteins. Branch lengths were extracted using a custom Python script (available at https://github.com/lstevens17/caeno-ten-descriptions). Table S8. EGF‐like repeat counts for LIN‐12/GLP‐1 homologues. Counts of EGF‐like repeats were obtained from were obtained from the ProSiteProfiles database (release 2017_09). Table S9. Accessions and links to data used in phylogenomic analysis. Table S10. Completeness and duplication statistics for 28 Caenorhabditis species based on 8,286 orthologues. We selected groups of orthologues which were present in at least 22 species and had a mean count of 1. The duplication ratio was calculated by dividing the total number of sequences present for each species by the total number of orthogroups which contained a representative sequence for that species. Figure S1. Assembly spans and genome size estimates. Kmers of length 19 were counted usi [file EVL3-3-217-s001.zip › evl3110-sup-0001-SuppMat/evl3110-sup-0007-FigureS1.pdf]

*Caenorhabditis parvicauda*

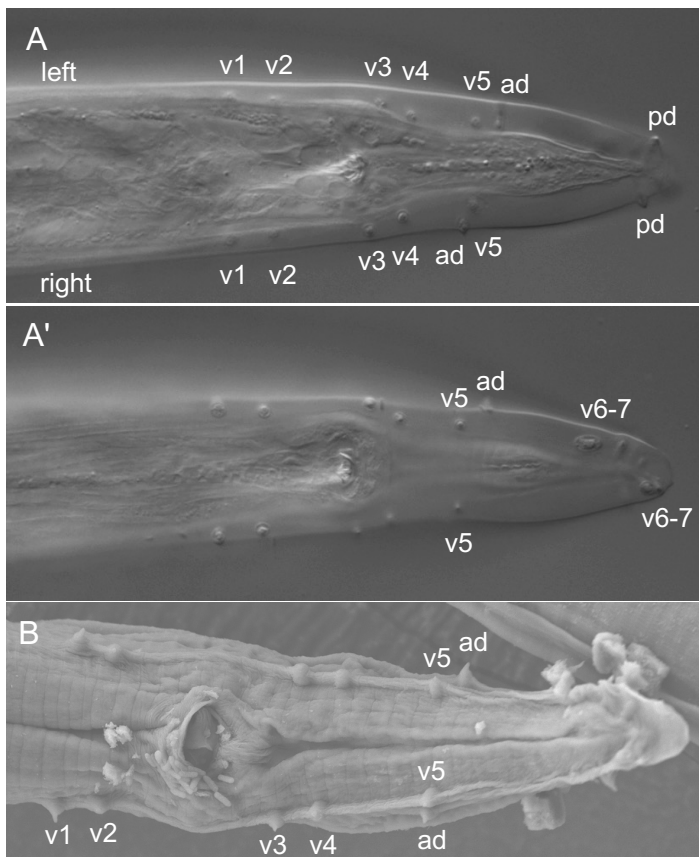

Supplement: Supplementary file 1 — Table S1. List of isolates and their origin. Table S2. Mating tests. This table contains several sheets, showing the results of crosses between isolates of different species. Successful crosses are labeled in green.“100s of embryos” refer to unhatched dead embryos remaining on the plate. Table S3. Detailed genome assembly and gene prediction statistics. Table S4. Morphological characters used for ancestral state reconstruction. ‘1’ denotes presence or existence; ‘0’ denotes absence. Table S5. Genome contents of C. sulstoni and C. elegans. Gene structure statistics were calculated using the longest isoform of each protein‐coding gene. UTR regions were not annotated in C. sulstoni and so were not considered in either species. Table S6. Genome statistics used in PGLS analysis. Gene structure statistics were calculated using the longest isoform of each protein‐coding gene. UTR regions were not considered as they were not annotated in several species. Repeat content was estimated de novo using RepeatModeler and RepeatMasker. Table S7. Mean branch lengths from Maximum likelihood gene tree of all Notch‐like proteins. Branch lengths were extracted using a custom Python script (available at https://github.com/lstevens17/caeno-ten-descriptions). Table S8. EGF‐like repeat counts for LIN‐12/GLP‐1 homologues. Counts of EGF‐like repeats were obtained from were obtained from the ProSiteProfiles database (release 2017_09). Table S9. Accessions and links to data used in phylogenomic analysis. Table S10. Completeness and duplication statistics for 28 Caenorhabditis species based on 8,286 orthologues. We selected groups of orthologues which were present in at least 22 species and had a mean count of 1. The duplication ratio was calculated by dividing the total number of sequences present for each species by the total number of orthogroups which contained a representative sequence for that species. Figure S1. Assembly spans and genome size estimates. Kmers of length 19 were counted usi [file EVL3-3-217-s001.zip › evl3110-sup-0001-SuppMat/evl3110-sup-0008-FigureS1.pdf]

*Caenorhabditis quiockensis*

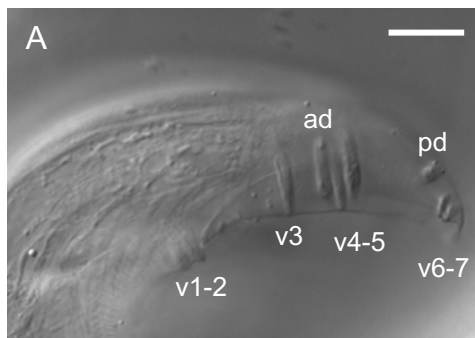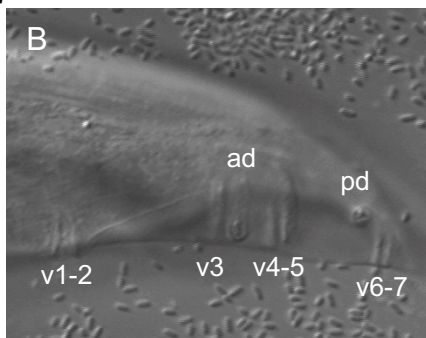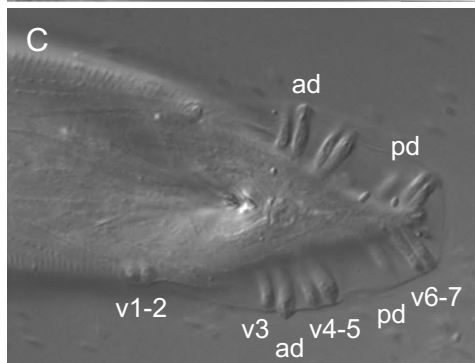

*Caenorhabditis castelli*

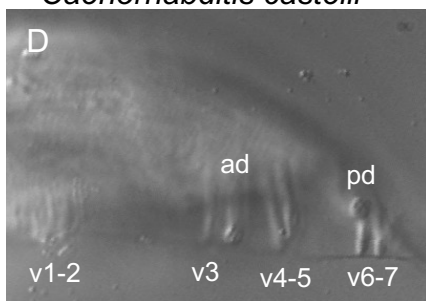

*Caenorhabditis vivipara*

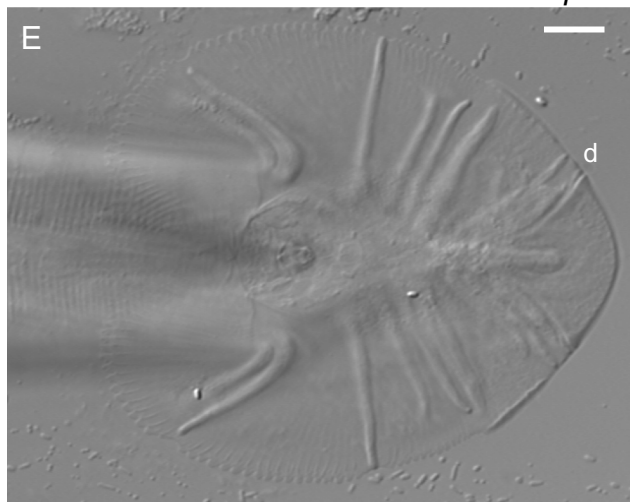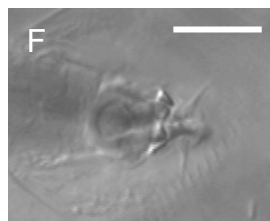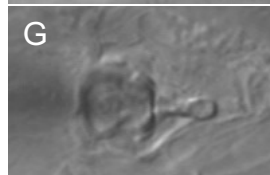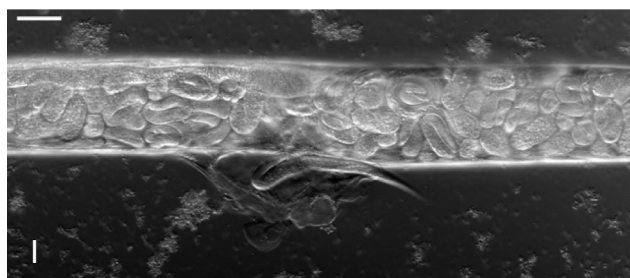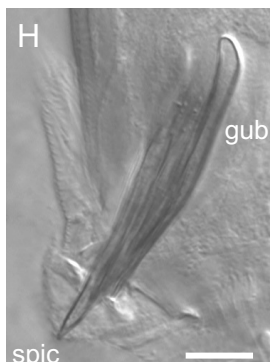

Supplement: Supplementary file 1 — Table S1. List of isolates and their origin. Table S2. Mating tests. This table contains several sheets, showing the results of crosses between isolates of different species. Successful crosses are labeled in green.“100s of embryos” refer to unhatched dead embryos remaining on the plate. Table S3. Detailed genome assembly and gene prediction statistics. Table S4. Morphological characters used for ancestral state reconstruction. ‘1’ denotes presence or existence; ‘0’ denotes absence. Table S5. Genome contents of C. sulstoni and C. elegans. Gene structure statistics were calculated using the longest isoform of each protein‐coding gene. UTR regions were not annotated in C. sulstoni and so were not considered in either species. Table S6. Genome statistics used in PGLS analysis. Gene structure statistics were calculated using the longest isoform of each protein‐coding gene. UTR regions were not considered as they were not annotated in several species. Repeat content was estimated de novo using RepeatModeler and RepeatMasker. Table S7. Mean branch lengths from Maximum likelihood gene tree of all Notch‐like proteins. Branch lengths were extracted using a custom Python script (available at https://github.com/lstevens17/caeno-ten-descriptions). Table S8. EGF‐like repeat counts for LIN‐12/GLP‐1 homologues. Counts of EGF‐like repeats were obtained from were obtained from the ProSiteProfiles database (release 2017_09). Table S9. Accessions and links to data used in phylogenomic analysis. Table S10. Completeness and duplication statistics for 28 Caenorhabditis species based on 8,286 orthologues. We selected groups of orthologues which were present in at least 22 species and had a mean count of 1. The duplication ratio was calculated by dividing the total number of sequences present for each species by the total number of orthogroups which contained a representative sequence for that species. Figure S1. Assembly spans and genome size estimates. Kmers of length 19 were counted usi [file EVL3-3-217-s001.zip › evl3110-sup-0001-SuppMat/evl3110-sup-0009-FigureS1.pdf]

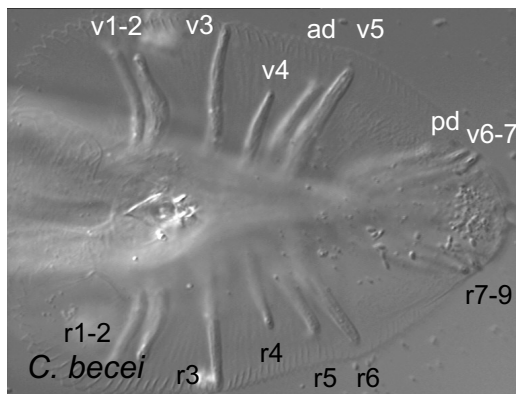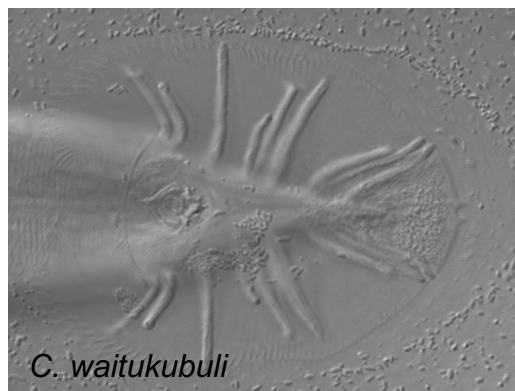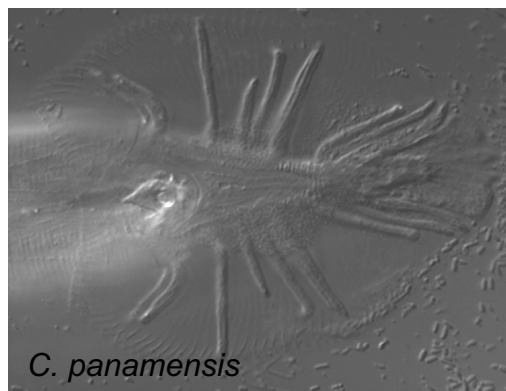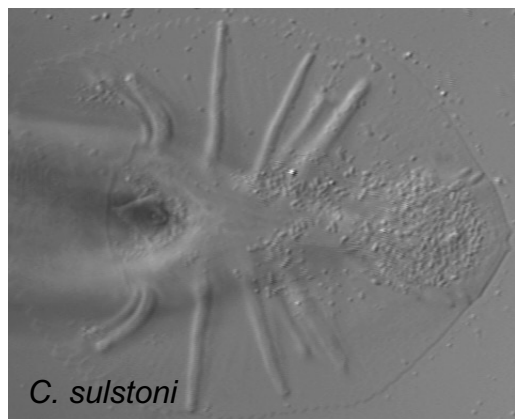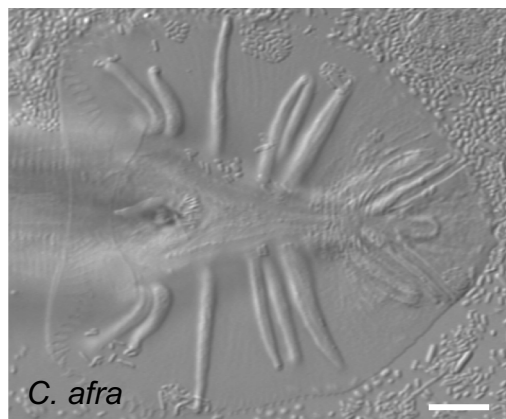

Supplement: Supplementary file 1 — Table S1. List of isolates and their origin. Table S2. Mating tests. This table contains several sheets, showing the results of crosses between isolates of different species. Successful crosses are labeled in green.“100s of embryos” refer to unhatched dead embryos remaining on the plate. Table S3. Detailed genome assembly and gene prediction statistics. Table S4. Morphological characters used for ancestral state reconstruction. ‘1’ denotes presence or existence; ‘0’ denotes absence. Table S5. Genome contents of C. sulstoni and C. elegans. Gene structure statistics were calculated using the longest isoform of each protein‐coding gene. UTR regions were not annotated in C. sulstoni and so were not considered in either species. Table S6. Genome statistics used in PGLS analysis. Gene structure statistics were calculated using the longest isoform of each protein‐coding gene. UTR regions were not considered as they were not annotated in several species. Repeat content was estimated de novo using RepeatModeler and RepeatMasker. Table S7. Mean branch lengths from Maximum likelihood gene tree of all Notch‐like proteins. Branch lengths were extracted using a custom Python script (available at https://github.com/lstevens17/caeno-ten-descriptions). Table S8. EGF‐like repeat counts for LIN‐12/GLP‐1 homologues. Counts of EGF‐like repeats were obtained from were obtained from the ProSiteProfiles database (release 2017_09). Table S9. Accessions and links to data used in phylogenomic analysis. Table S10. Completeness and duplication statistics for 28 Caenorhabditis species based on 8,286 orthologues. We selected groups of orthologues which were present in at least 22 species and had a mean count of 1. The duplication ratio was calculated by dividing the total number of sequences present for each species by the total number of orthogroups which contained a representative sequence for that species. Figure S1. Assembly spans and genome size estimates. Kmers of length 19 were counted usi [file EVL3-3-217-s001.zip › evl3110-sup-0001-SuppMat/evl3110-sup-0010-FigureS1.pdf]

**A****Dorsal ray position**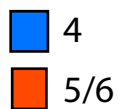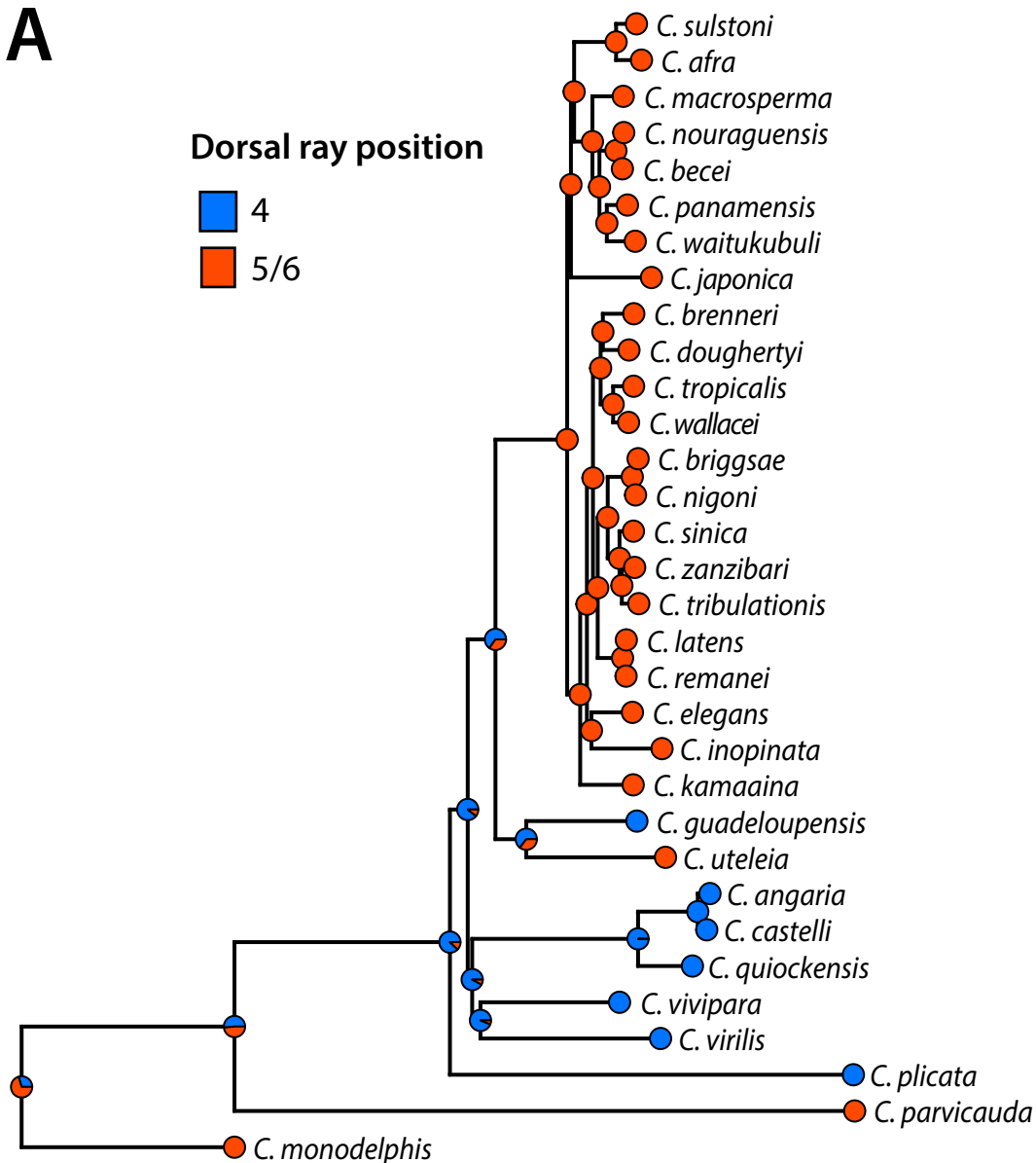**B****Closed fan**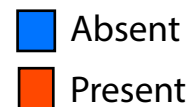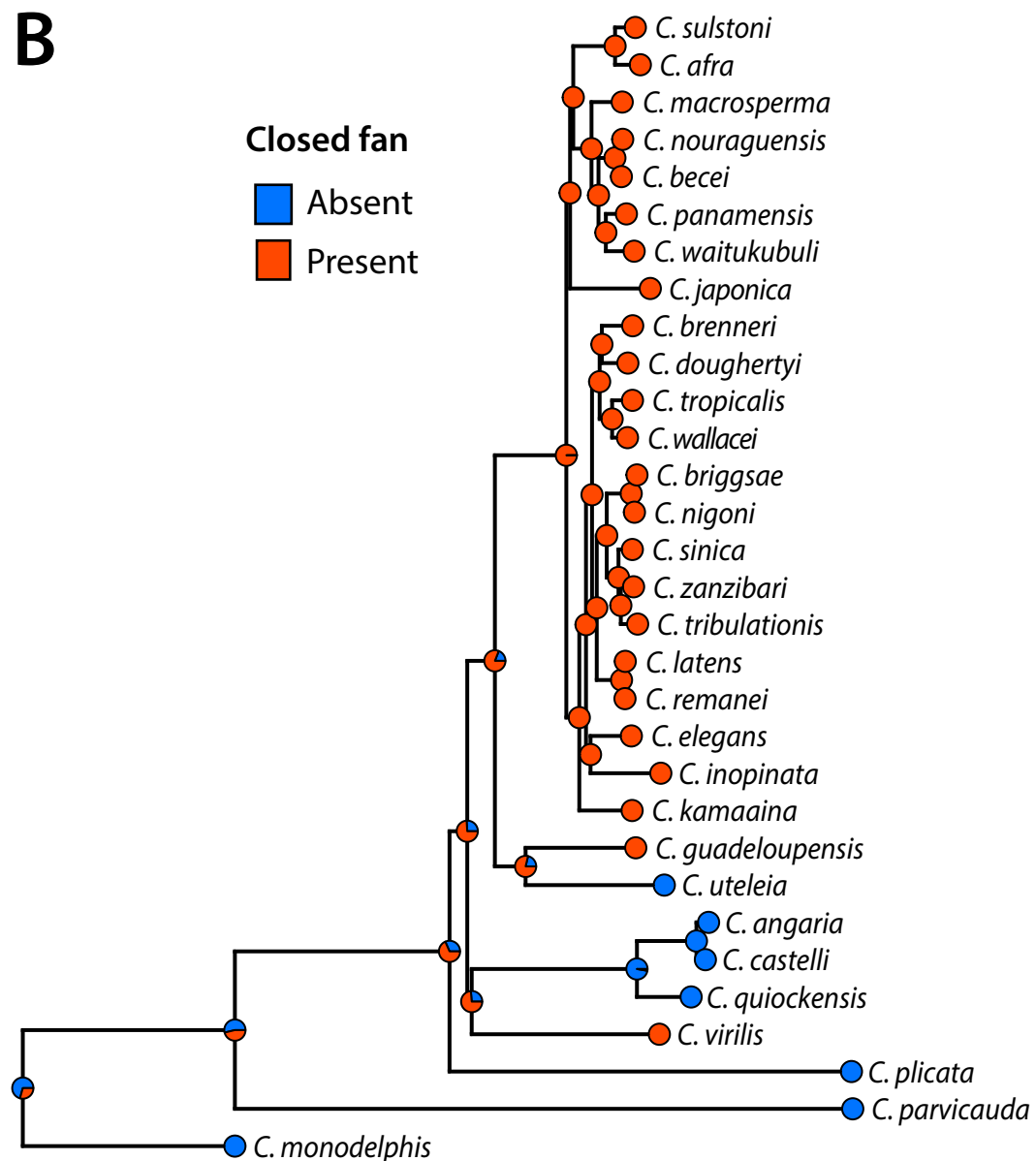

Supplement: Supplementary file 1 — Table S1. List of isolates and their origin. Table S2. Mating tests. This table contains several sheets, showing the results of crosses between isolates of different species. Successful crosses are labeled in green.“100s of embryos” refer to unhatched dead embryos remaining on the plate. Table S3. Detailed genome assembly and gene prediction statistics. Table S4. Morphological characters used for ancestral state reconstruction. ‘1’ denotes presence or existence; ‘0’ denotes absence. Table S5. Genome contents of C. sulstoni and C. elegans. Gene structure statistics were calculated using the longest isoform of each protein‐coding gene. UTR regions were not annotated in C. sulstoni and so were not considered in either species. Table S6. Genome statistics used in PGLS analysis. Gene structure statistics were calculated using the longest isoform of each protein‐coding gene. UTR regions were not considered as they were not annotated in several species. Repeat content was estimated de novo using RepeatModeler and RepeatMasker. Table S7. Mean branch lengths from Maximum likelihood gene tree of all Notch‐like proteins. Branch lengths were extracted using a custom Python script (available at https://github.com/lstevens17/caeno-ten-descriptions). Table S8. EGF‐like repeat counts for LIN‐12/GLP‐1 homologues. Counts of EGF‐like repeats were obtained from were obtained from the ProSiteProfiles database (release 2017_09). Table S9. Accessions and links to data used in phylogenomic analysis. Table S10. Completeness and duplication statistics for 28 Caenorhabditis species based on 8,286 orthologues. We selected groups of orthologues which were present in at least 22 species and had a mean count of 1. The duplication ratio was calculated by dividing the total number of sequences present for each species by the total number of orthogroups which contained a representative sequence for that species. Figure S1. Assembly spans and genome size estimates. Kmers of length 19 were counted usi [file EVL3-3-217-s001.zip › evl3110-sup-0001-SuppMat/evl3110-sup-0011-FigureS1.pdf]

# Spicule tip shape

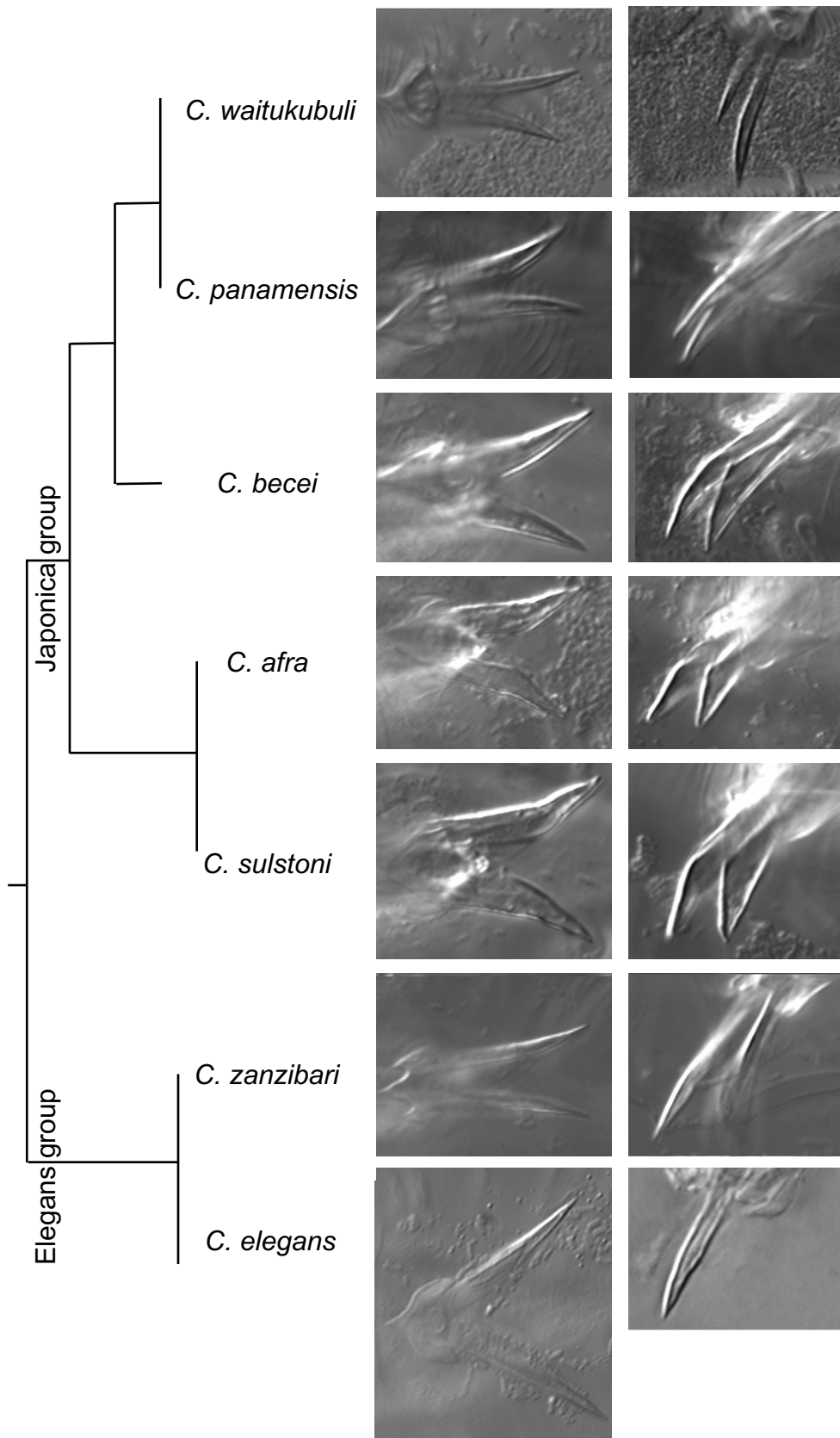

Supplement: Supplementary file 1 — Table S1. List of isolates and their origin. Table S2. Mating tests. This table contains several sheets, showing the results of crosses between isolates of different species. Successful crosses are labeled in green.“100s of embryos” refer to unhatched dead embryos remaining on the plate. Table S3. Detailed genome assembly and gene prediction statistics. Table S4. Morphological characters used for ancestral state reconstruction. ‘1’ denotes presence or existence; ‘0’ denotes absence. Table S5. Genome contents of C. sulstoni and C. elegans. Gene structure statistics were calculated using the longest isoform of each protein‐coding gene. UTR regions were not annotated in C. sulstoni and so were not considered in either species. Table S6. Genome statistics used in PGLS analysis. Gene structure statistics were calculated using the longest isoform of each protein‐coding gene. UTR regions were not considered as they were not annotated in several species. Repeat content was estimated de novo using RepeatModeler and RepeatMasker. Table S7. Mean branch lengths from Maximum likelihood gene tree of all Notch‐like proteins. Branch lengths were extracted using a custom Python script (available at https://github.com/lstevens17/caeno-ten-descriptions). Table S8. EGF‐like repeat counts for LIN‐12/GLP‐1 homologues. Counts of EGF‐like repeats were obtained from were obtained from the ProSiteProfiles database (release 2017_09). Table S9. Accessions and links to data used in phylogenomic analysis. Table S10. Completeness and duplication statistics for 28 Caenorhabditis species based on 8,286 orthologues. We selected groups of orthologues which were present in at least 22 species and had a mean count of 1. The duplication ratio was calculated by dividing the total number of sequences present for each species by the total number of orthogroups which contained a representative sequence for that species. Figure S1. Assembly spans and genome size estimates. Kmers of length 19 were counted usi [file EVL3-3-217-s001.zip › evl3110-sup-0001-SuppMat/evl3110-sup-0012-FigureS1.pdf]

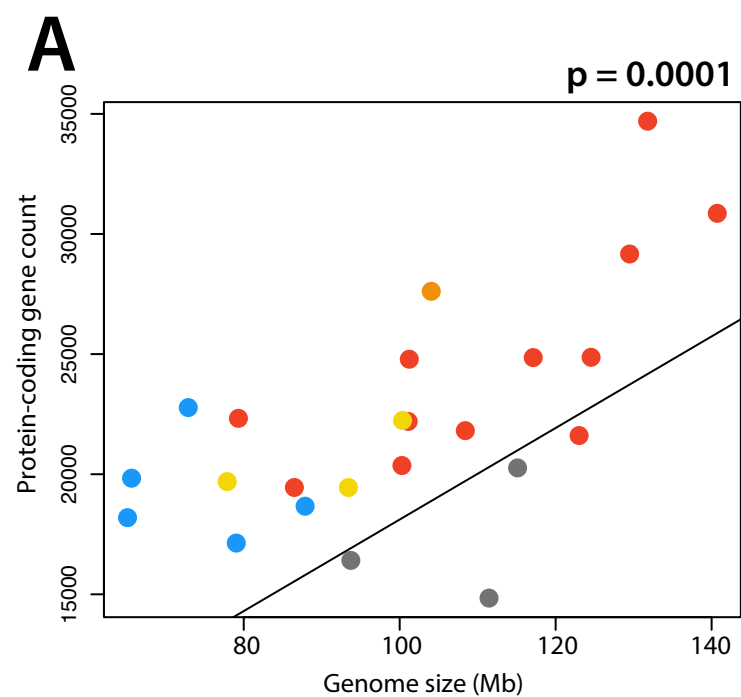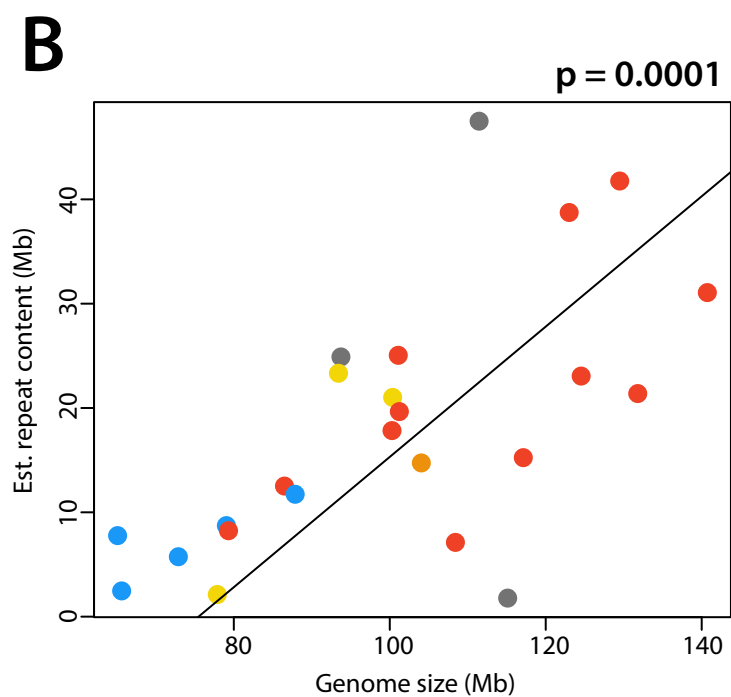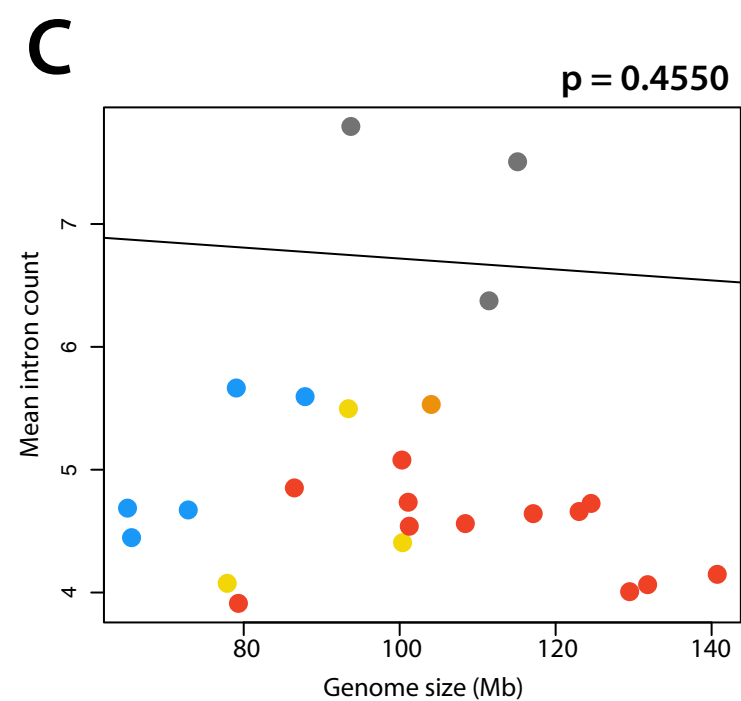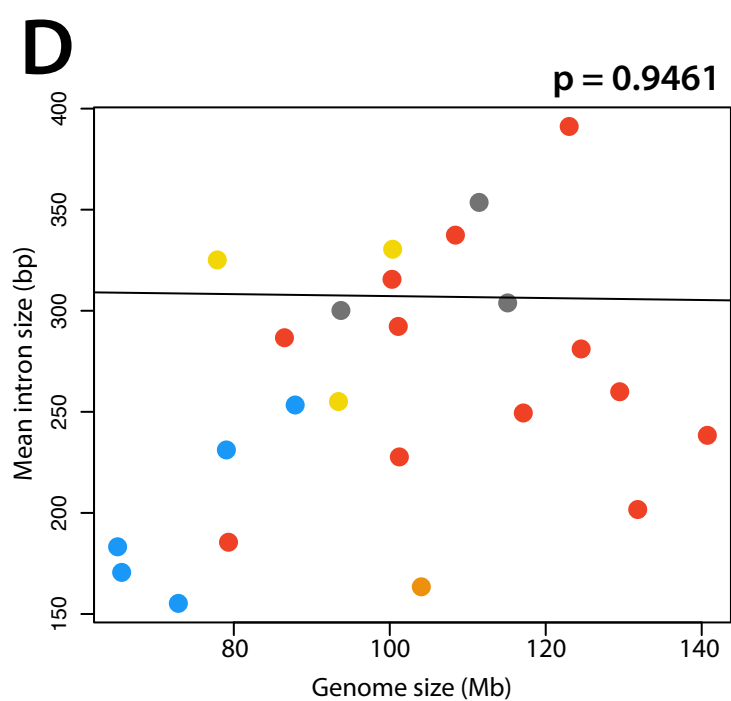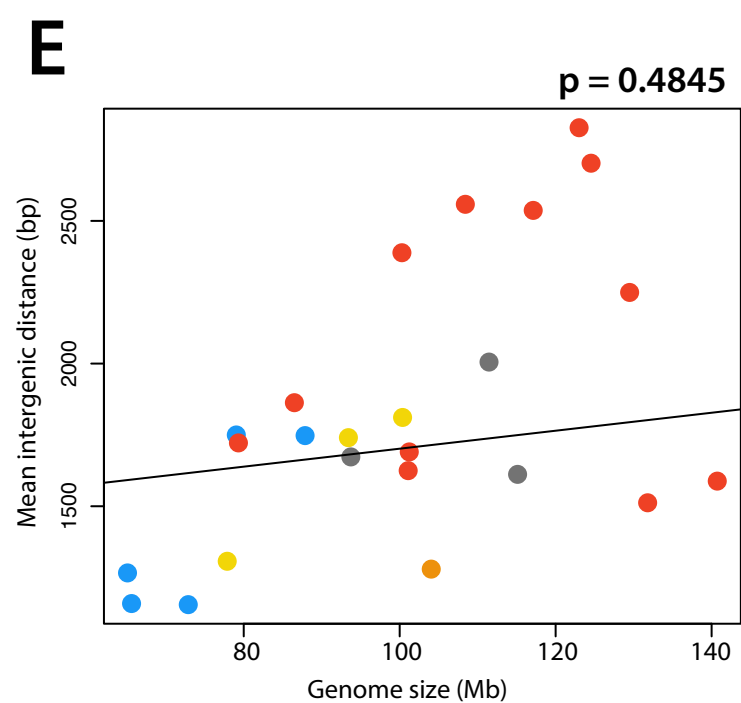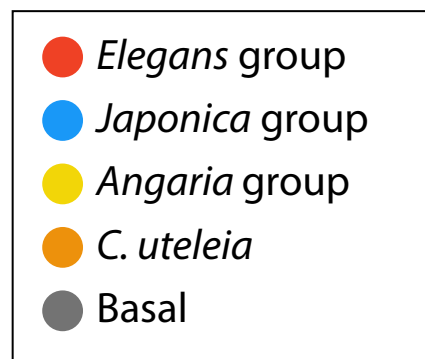

Supplement: Supplementary file 1 — Table S1. List of isolates and their origin. Table S2. Mating tests. This table contains several sheets, showing the results of crosses between isolates of different species. Successful crosses are labeled in green.“100s of embryos” refer to unhatched dead embryos remaining on the plate. Table S3. Detailed genome assembly and gene prediction statistics. Table S4. Morphological characters used for ancestral state reconstruction. ‘1’ denotes presence or existence; ‘0’ denotes absence. Table S5. Genome contents of C. sulstoni and C. elegans. Gene structure statistics were calculated using the longest isoform of each protein‐coding gene. UTR regions were not annotated in C. sulstoni and so were not considered in either species. Table S6. Genome statistics used in PGLS analysis. Gene structure statistics were calculated using the longest isoform of each protein‐coding gene. UTR regions were not considered as they were not annotated in several species. Repeat content was estimated de novo using RepeatModeler and RepeatMasker. Table S7. Mean branch lengths from Maximum likelihood gene tree of all Notch‐like proteins. Branch lengths were extracted using a custom Python script (available at https://github.com/lstevens17/caeno-ten-descriptions). Table S8. EGF‐like repeat counts for LIN‐12/GLP‐1 homologues. Counts of EGF‐like repeats were obtained from were obtained from the ProSiteProfiles database (release 2017_09). Table S9. Accessions and links to data used in phylogenomic analysis. Table S10. Completeness and duplication statistics for 28 Caenorhabditis species based on 8,286 orthologues. We selected groups of orthologues which were present in at least 22 species and had a mean count of 1. The duplication ratio was calculated by dividing the total number of sequences present for each species by the total number of orthogroups which contained a representative sequence for that species. Figure S1. Assembly spans and genome size estimates. Kmers of length 19 were counted usi [file EVL3-3-217-s001.zip › evl3110-sup-0001-SuppMat/evl3110-sup-0013-FigureS1.pdf]

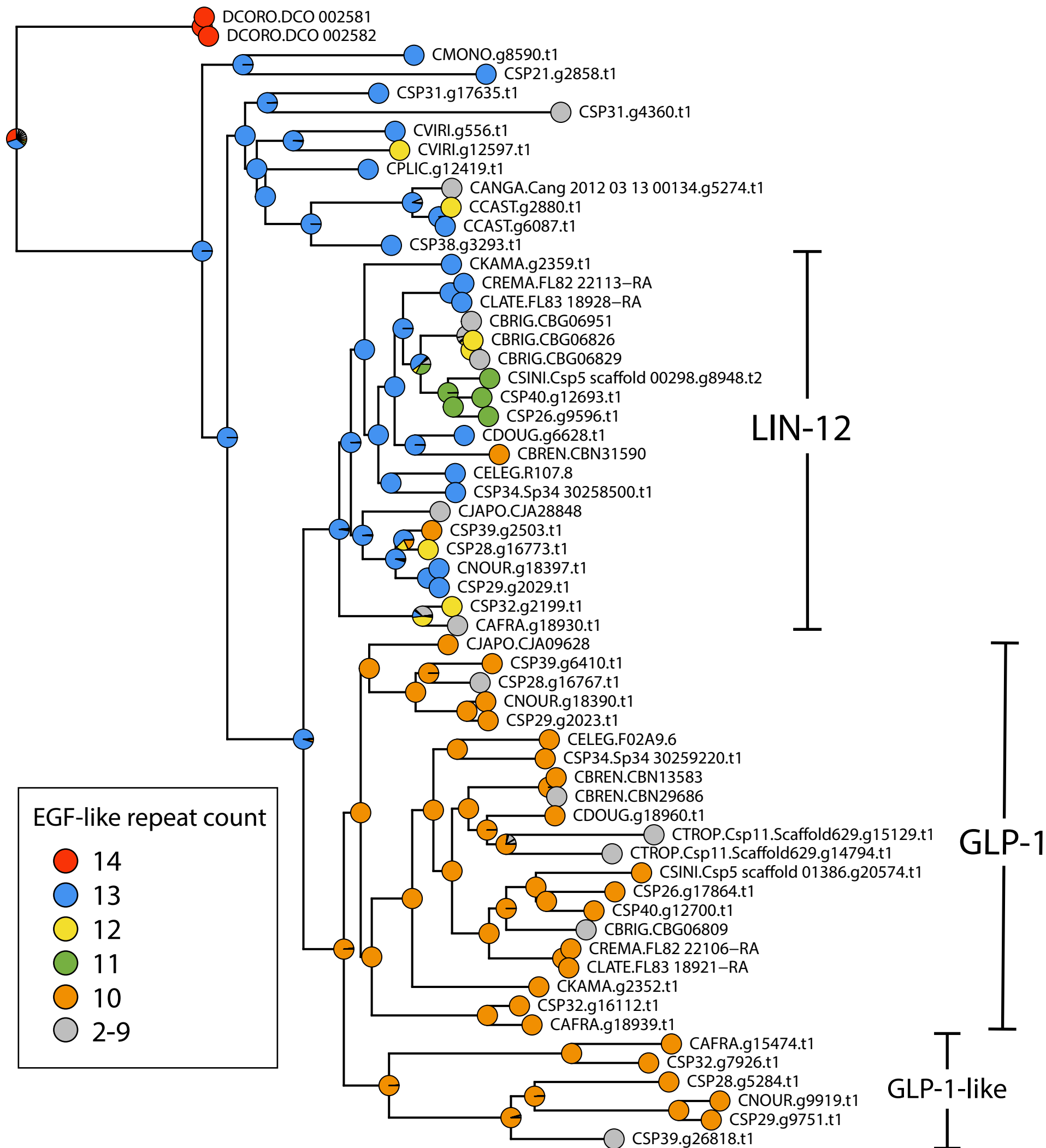

Supplement: Supplementary file 1 — Table S1. List of isolates and their origin. Table S2. Mating tests. This table contains several sheets, showing the results of crosses between isolates of different species. Successful crosses are labeled in green.“100s of embryos” refer to unhatched dead embryos remaining on the plate. Table S3. Detailed genome assembly and gene prediction statistics. Table S4. Morphological characters used for ancestral state reconstruction. ‘1’ denotes presence or existence; ‘0’ denotes absence. Table S5. Genome contents of C. sulstoni and C. elegans. Gene structure statistics were calculated using the longest isoform of each protein‐coding gene. UTR regions were not annotated in C. sulstoni and so were not considered in either species. Table S6. Genome statistics used in PGLS analysis. Gene structure statistics were calculated using the longest isoform of each protein‐coding gene. UTR regions were not considered as they were not annotated in several species. Repeat content was estimated de novo using RepeatModeler and RepeatMasker. Table S7. Mean branch lengths from Maximum likelihood gene tree of all Notch‐like proteins. Branch lengths were extracted using a custom Python script (available at https://github.com/lstevens17/caeno-ten-descriptions). Table S8. EGF‐like repeat counts for LIN‐12/GLP‐1 homologues. Counts of EGF‐like repeats were obtained from were obtained from the ProSiteProfiles database (release 2017_09). Table S9. Accessions and links to data used in phylogenomic analysis. Table S10. Completeness and duplication statistics for 28 Caenorhabditis species based on 8,286 orthologues. We selected groups of orthologues which were present in at least 22 species and had a mean count of 1. The duplication ratio was calculated by dividing the total number of sequences present for each species by the total number of orthogroups which contained a representative sequence for that species. Figure S1. Assembly spans and genome size estimates. Kmers of length 19 were counted usi [file EVL3-3-217-s001.zip › evl3110-sup-0001-SuppMat/evl3110-sup-0014-FigureS1.pdf]
